# SAR11 Cells Rely on Enzyme Multifunctionality To Metabolize a Range of Polyamine Compounds

**DOI:** 10.1128/mBio.01091-21

**Published:** 2021-08-24

**Authors:** Stephen E. Noell, Gregory E. Barrell, Christopher Suffridge, Jeff Morré, Kevin P. Gable, Jason R. Graff, Brian J. VerWey, Ferdi L. Hellweger, Stephen J. Giovannoni

**Affiliations:** a Department of Microbiology, Oregon State University, Corvallis, Oregon, USA; b Department of Chemistry, Oregon State University, Corvallis, Oregon, USA; c Department of Botany & Plant Pathology, Oregon State University, Corvallis, Oregon, USA; d Water Quality Engineering, Berlin TU, Berlin, Germany; Max Planck Institute for Marine Microbiology

**Keywords:** SAR11, marine microbiology, metabolism, physiology, polyamines

## Abstract

In the ocean surface layer and cell culture, the polyamine transport protein PotD of SAR11 bacteria is often one of the most abundant proteins detected. Polyamines are organic cations at seawater pH produced by all living organisms and are thought to be an important component of dissolved organic matter (DOM) produced in planktonic ecosystems. We hypothesized that SAR11 cells uptake and metabolize multiple polyamines and use them as sources of carbon and nitrogen. Metabolic footprinting and fingerprinting were used to measure the uptake of five polyamine compounds (putrescine, cadaverine, agmatine, norspermidine, and spermidine) in two SAR11 strains that represent the majority of SAR11 cells in the surface ocean environment, “*Candidatus* Pelagibacter” strain HTCC7211 and “*Candidatus* Pelagibacter ubique” strain HTCC1062. Both strains took up all five polyamines and concentrated them to micromolar or millimolar intracellular concentrations. Both strains could use most of the polyamines to meet their nitrogen requirements, but polyamines did not fully substitute for their requirements of glycine (or related compounds) or pyruvate (or related compounds). Our data suggest that potABCD transports all five polyamines and that spermidine synthase, speE, is reversible, catalyzing the breakdown of spermidine and norspermidine, in addition to its usual biosynthetic role. These findings provide support for the hypothesis that enzyme multifunctionality enables streamlined cells in planktonic ecosystems to increase the range of DOM compounds they metabolize.

## INTRODUCTION

Polyamines are low-molecular-weight organic polycations that are ubiquitous in living organisms. They play a role in stabilizing DNA, RNA, and proteins, are required for cell growth, and have been implicated in biofilm formation (1–3). Polyamine compounds and concentrations vary between cell types and can depend on nutrient status, temperature, and salinity (4). Polyamines are found at low nanomolar concentrations in the coastal and open ocean, reaching maximal concentrations of 30 nM during algal blooms, but typically are around 1 nM (5–7). Polyamines from the environment are metabolized by bacteria as nitrogen and carbon sources at rates similar to those of dissolved free amino acids and supply up to 14% of bacterial nitrogen demand in coastal regions (8–10).

**FIG 1:**
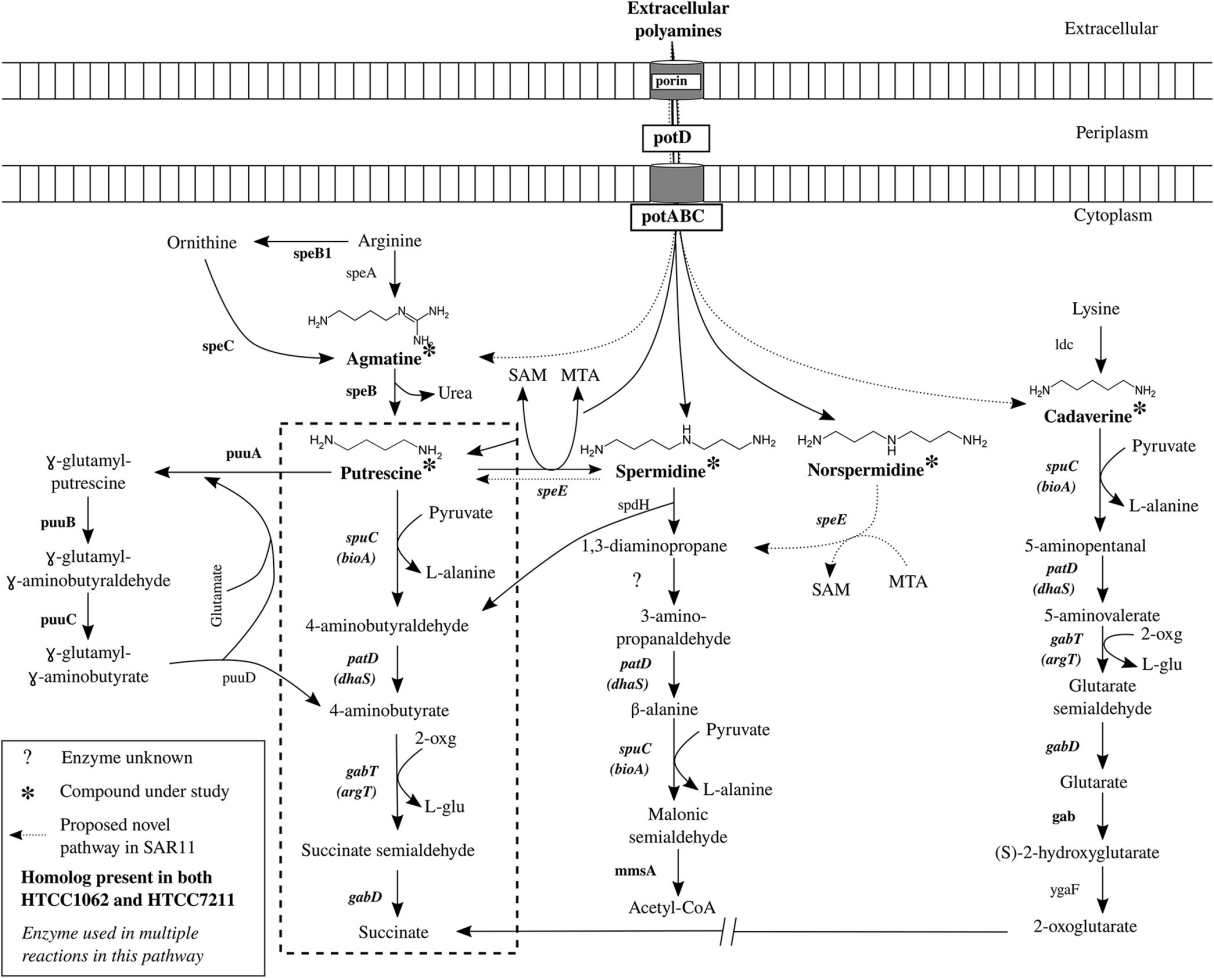
Polyamine compound metabolism in SAR11. Common pathways for polyamine metabolism in bacteria are shown. The compounds under study are marked with asterisks, with enzymes listed in bold if both strains of SAR11 in this study, HTCC1062 and HTCC7211, have homologs to the enzyme, or in plain text if in neither. A question mark indicates that the enzyme is unknown. The enzyme name is in italics if it is used for multiple reactions in this metabolic system. The dashed box encompasses the pathway thought to be used by SAR11 cells for PUT metabolism based on previous studies. By-products of reactions where NH_3_ groups are transferred are included to show the flow of N. Gene names for SAR11 homologs, where different from canonical gene names are listed below the canonical name in parentheses. *potC*, spermidine/putrescine ABC transporter, permease; *potB*, permease; *potD*, SBP; *potA*, ATP-binding protein; *speB1*, arginase; *speC*, lysine/ornithine decarboxylase; *speB*, agmatinase; *speE*, spermidine/spermine synthase; *puuA*, gamma-glutamylputrescine synthetase; *puuB*, gamma-glutamylputrescine oxidoreductase; *puuC*, NADP/NAD-dependent aldehyde dehydrogenase; *puuD*, gamma-glutamyl-gamma-aminobutyrate hydrolase; *spuC*, putrescine-pyruvate aminotransferase; *patD*, 4-aminobutyraldehyde dehydrogenase; *gabT*, acetylornithine aminotransferase; *gabD*, succinate-semialdehyde dehydrogenase; *spdH*, spermidine dehydrogenase; *mmsA*, malonate-semialdehyde dehydrogenase; *gab*, glutarate 2-hydroxylase; *ygaF*, l-2-hydroxyglutarate dehydrogenase. SAM; *S*-adenosylmethionine; MTA, 5′-methylthioadenosine; 2-oxg, 2-oxoglutarate; l-glu, l-glutamate.

Putrescine (PUT) and spermidine (SPD), the most abundant polyamines in the oceanic dissolved pool, are typically 3 to 5 nM in the environment, but spermine, cadaverine (CAD), and norspermidine (NSD) have been detected at lower levels (6, 10, 11). Several other polyamines, such as 1,3-diaminopropane (DAP), agmatine (AGM), homospermidine (HSD), spermine, and larger, more complex polyamines are known to be produced and/or metabolized by cells from all domains of life (3, 4, 9, 12, 13). Metabolic pathways for common polyamines are shown in Fig. 1.

SAR11 alphaproteobacteria make up the majority of bacteria in the ocean (14). SAR11 cells primarily utilize labile, low-molecular-weight molecules (15). They pack their relatively large periplasmic space (16) with large numbers of ABC transporter substrate-binding proteins (SBPs) (17, 18), increasing the encounter rate and binding of substrate molecules with SBPs, resulting in high whole-cell uptake affinities (19, 20). Recent modeling work has extended this observation, suggesting that this strategy may contribute to the low growth rates of SAR11 cells (21). SAR11 bacteria evolved minimal genomes in response to streamlining selection, which favors efficient use of resources in nutrient-limited ecosystems (22). Enzyme multifunctionality, defined broadly as enzymes that are adapted to carry out more than one function (23, 24), and particularly enzymes that are adapted to have broader substrate and catalytic range than normal, has been hypothesized to reduce gene content in streamlined cells and has been confirmed for the SAR11 glycine betaine transporter (20). Here, we use multifunctionality rather than promiscuity to differentiate enzymes that interact with multiple substrates at a single catalytic site in response to evolutionary pressure (25). This retains promiscuity for describing enzyme interactions with substrates that are not under evolutionary pressure.

SAR11 cells produce large numbers of PotD, the SBP involved in polyamine transport, both in cultures and the environment, making it the most highly expressed transporter for N-related compounds by SAR11 cells (17, 18, 26). N-limited cultures of SAR11 strain HTCC1062, a member of the cold, high-latitude group Ia.1 ecotype, did not upregulate genes for polyamine transport or metabolism, except for an enzyme implicated in PUT and CAD metabolism (27), but genes involved in the metabolism and transport of other organic N sources were upregulated (27). Incubation experiments with natural seawater communities provided evidence that SAR11 cells may sometimes respond to additions of the polyamines PUT and SPD; transcripts for SAR11 genes involved in polyamine metabolism increased in the first hour of incubation and accounted for over a quarter of all transcripts (28). In other experiments with PUT and SPD amendments to seawater, it was observed that SAR11 cell abundance did not change during a 48-h period in response to PUT and SPD addition (29, 30); oligotrophs frequently decrease in relative abundance in incubation experiments due to their low growth rates, while copiotrophs increase rapidly due to their high growth rates under high nutrient conditions used in incubation experiments (15).

In this study, we used targeted metabolic footprinting and fingerprinting to examine the types and amounts of polyamines taken up and metabolized by two SAR11 strains. Both strains of SAR11 used in this study come from the Ia subgroup; “*Candidatus* Pelagibacter ubique” HTCC1062 belongs to the cold, high-latitude group Ia.1 ecotype, and “*Candidatus* Pelagibacter” strain HTCC7211 is from the equatorial, warm water Ia.3 ecotype (15). Targeted metabolic footprinting uses liquid chromatography tandem mass spectrometry (LC-MS/MS) to measure changes in the concentrations of specific metabolites dissolved in spent culture media (31), while fingerprinting quantifies the concentrations of targeted metabolites within cells (32). We hypothesized that SAR11 cells would use polyamines as N sources and that polyamines might be substitutes for their conditional auxotrophic requirement of pyruvate (28, 33).

## RESULTS

### Footprinting and fingerprinting experiments.

We focused on five polyamine compounds (Table 1), putrescine (PUT), cadaverine (CAD), agmatine (AGM), norspermidine (NSD), and spermidine (SPD). These compounds were picked either for their prevalence in the environment and in bacterial cells or for their role as precursors to other polyamine compounds (4, 6, 34, 35). These compounds also showed the best recovery in solid-phase extraction and were amenable to simultaneous quantification by LC-MS/MS. We used polyamine concentrations of 10- to 100-fold ambient environmental concentrations, similar to what would be found in nutrient patches (36), as has been done previously (20, 28). In preliminary experiments, we found that high polyamine concentrations inhibited growth; HTCC1062 growth was inhibited when all polyamines were added together at individual concentrations above 500 nM, while HTCC7211 growth was inhibited at concentrations above 250 nM. We chose 500 nM for HTCC1062 and 250 nM for HTCC7211, the highest concentrations that did not significantly inhibit growth, for further experiments (Fig. S1A and B).

**TABLE 1:** List of compounds used in the project as well as LC-MS/MS parameters^*a*^

Compound (abbreviation)	Structure	Mol wt (g/ mol)	Retention time (min)	MRM parent product *m/z*^*b*^	LOD (nM)
1,3-Diamino-propane (DAP)	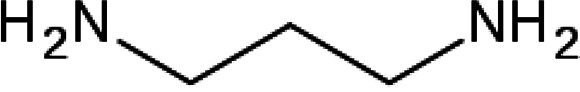	74.1	1.90	75.1–58.1	4.51
Putrescine (PUT)	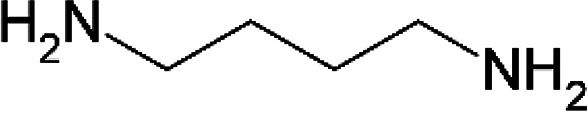	88.2	1.92	89.1–72	2.29
Cadaverine (CAD)	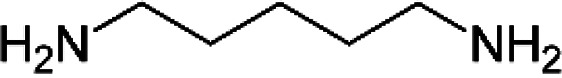	102.2	1.95	103.1– 69, 86	1.29
Agmatine (AGM)	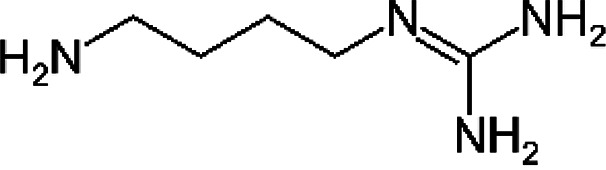	130.2	2.01	131.2–72, 60	1.85
Norspermidine (NSD)	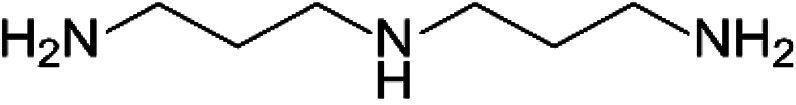	131.2	1.74	132.2–115, 98	2.06
Spermidine (SPD)	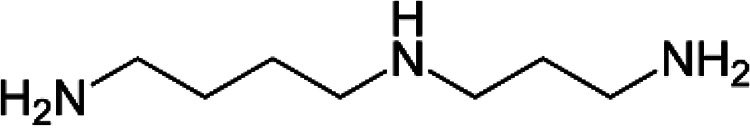	145.3	1.75	146.1–112.1, 72	1.25
^13^C-spermidine (IS)	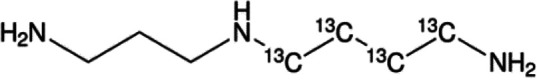	149.2	1.78	150.2–116.1, 76.1	
Homospermidine (HSD)	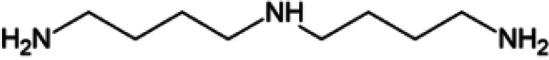	159.3	1.71	160.2–126.0, 83.8	5.58

*^a^*The instrumental limit of detection (LOD) for the LC-MS/MS was calculated as three times the standard deviation of the lowest detectable concentration of standards used (5 nM). The actual LOD of samples varied based on how much the samples were concentrated but was generally 2 to 1,000-fold lower than the instrumental LOD. A collision energy of 20 eV was used for all analytes except 1,3-diaminopropane, which had a CE of 10 eV, and homospermidine, which had a CE of 30 eV.

*^b^*The first listed product *m/z* was used for quantification; the second was used for verification. In cases where no second product is listed, the other products were too small for accurate identification.

10.1128/mBio.01091-21.6 FIG S1 (A and B) Growth experiments were carried out to determine the maximum concentration of polyamine compounds that can be added without negatively affecting growth of either (A) “*Ca.* Pelagibacter ubique” HTCC1062 or (B) “*Ca.* Pelagibacter” strain HTCC7211 under nutrient-replete conditions (see main text, Materials and Methods, for media used). Compounds were added at final concentrations of either 0 (control), 100, 250, 500, or 1,000 nM for each compound. Error bars are the standard deviation of triplicate samples. (C and D) Cell count data from the cultures used for footprinting experiments for either (C) HTCC1062 or (D) HTCC7211. The treatments with polyamines (PA) added had either a 500 nM (HTCC1062) or 250 nM (HTCC7211) final concentration of each of the five polyamines added. Error bars are the standard deviation of quadruplicate samples. (E and F) Cell count data from the cultures used in experiments to measure for the presence of 1,3-diaminopropane and homospermidine in either (E) HTCC1062 or (F) HTCC7211. The treatments with norspermidine (NSD) and spermidine (SPD) added had a 500 nM final concentration of each added. Error bars are the standard deviation of quadruplicate samples. (G) Cell count data from an experiment to monitor changes in cell size and morphology in response to polyamine addition in HTCC7211 cells using flow cytometry. Control, no polyamines added; early addition, 250 nM of each polyamine compound was added at the beginning of growth; late addition, 250 nM of each polyamine compound was added after 4 days of growth. Error bars are the standard deviations of quadruplicate cultures. Download FIG S1, PDF file, 0.2 MB. Copyright © 2021 Noell et al. 2021 Noell et al. https://creativecommons.org/licenses/by/4.0/ This content is distributed under the terms of the Creative Commons Attribution 4.0 International license.

**FIG 2:**
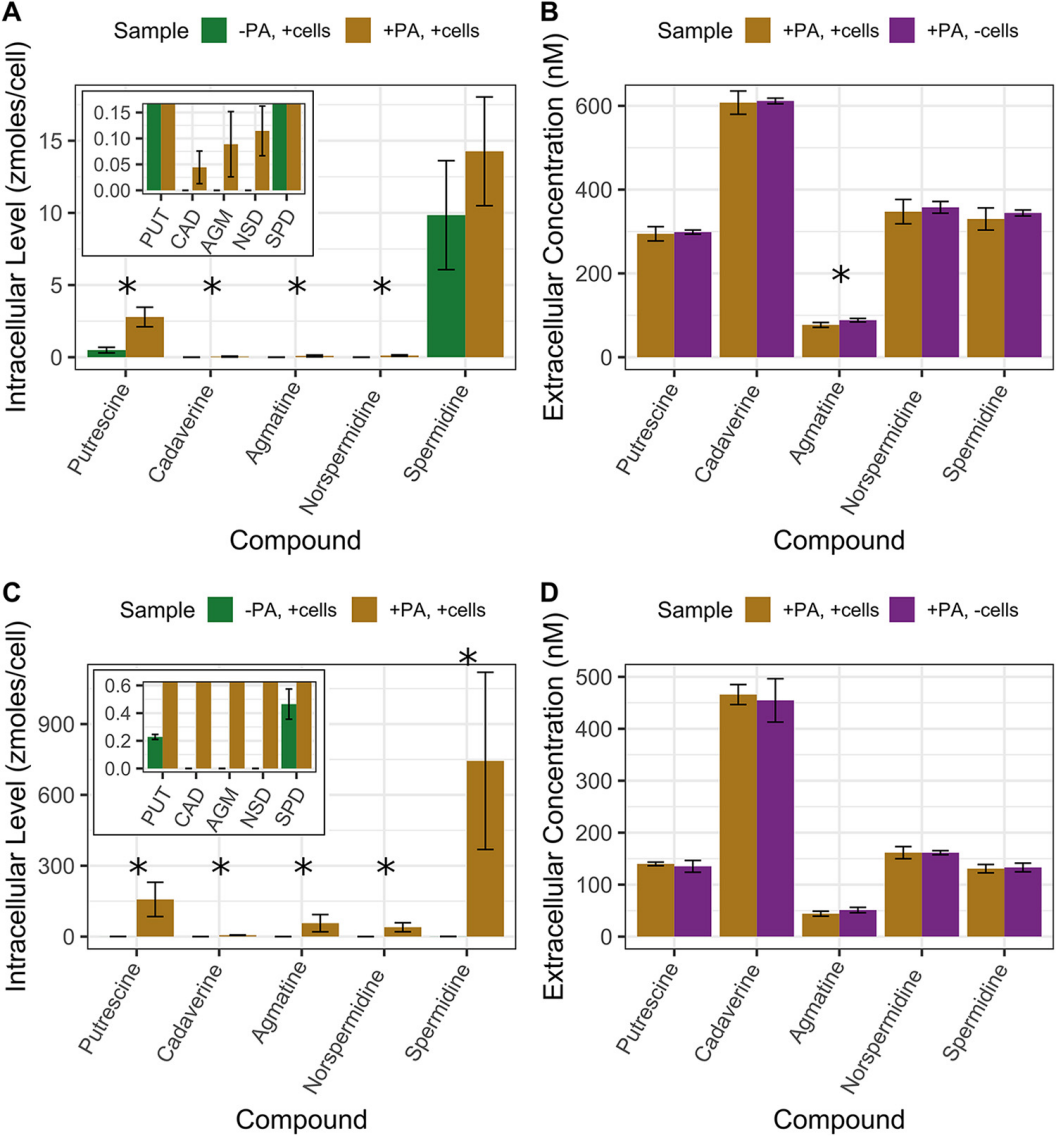
(A to D) Results of footprinting/fingerprinting experiments with (A and B) “*Ca*. Pelagibacter ubique” HTCC1062 and (C and D) “*Ca*. Pelagibacter” strain HTCC7211 with five polyamine compounds. (A and C) Intracellular concentrations of polyamine compounds in cultures with no polyamines added or with a 500 nM or 250 nM final concentration of each polyamine compound added for HTCC1062 or HTCC7211, respectively. (B and D) Extracellular concentrations of polyamine compounds in cultures with a 500 nM or 250 nM final concentration of each polyamine compound added for HTCC1062 or HTCC7211, respectively. The insets in panels A and C show compounds that had very low levels. Error bars are the standard deviations of quadruplicate cultures. * indicates a significant difference (*P* < 0.05, one-tailed *t* test) between the two treatments; for panels A and C, it indicates a significantly higher level of that compound in the experimental treatment compared to the negative control; for panels B and D, it indicates a significantly lower concentration of that compound in the experimental treatment compared to the no-cells control; for *P* values, see Table 2.

The five polyamines were added to SAR11 cultures under nutrient-replete conditions to measure uptake and metabolism of these compounds. Cultures were grown to late exponential phase before harvesting; growth rates were slightly lower with polyamines added—0.44 compared to 0.46 day^−1^ for HTCC1062 and 0.50 compared to 0.60 day^−1^ for HTCC7211 with and without polyamines (Fig. S2C and D). For both strains, the average intracellular levels of all five polyamine compounds were significantly greater in the experimental treatment (polyamines added) than in the negative control (no polyamines added), except for SPD in HTCC1062, which had nonsignificantly higher levels in experimental cultures (Fig. 2A and C; *P* values in Table 2). When the intracellular levels are converted to intracellular concentrations using a cell volume of 0.03 μm^3^ for HTCC1062 (16) and 0.04 μm^3^ for HTCC7211 (37), it is apparent that the cells are concentrating all compounds into intracellular concentrations greater than their environment (Table 2). The intracellular concentrations in the experimental treatment for HTCC7211 were much higher than those in HTCC1062, especially SPD, which was 40 times higher.

**TABLE 2:** Intracellular concentrations of polyamine compounds in SAR11 fingerprinting experiments (see Fig. 2)^*a*^

Compound	HTCC1062 intracellular concn (μM)			HTCC7211 intracellular concn (μM)		
	Neg. Control	+Polyamines	*P* value	Neg. Control	+ Polyamines	*P* value
Putrescine	16 ± 6.7	93 ± 23	0.003	6 ± 0.3	4,030 ± 1,860	0.03
Cadaverine	0	1.5 ± 1	0.03	0	153 ± 31	0.0002
Agmatine	0	3 ± 2.1	0.03	0	1,460 ± 940	0.02
Nor-spermidine	0	3.8 ± 1.6	0.01	0	1,020 ± 490	0.003
Spermidine	328 ± 127	480 ± 130	0.19	12 ± 2.7	19,100 ± 9,630	0.01

a Concentrations were calculated using cell volumes of 0.03 and 0.04 μm^3^ for HTCC1062 and HTCC7211, respectively. The listed *P* values are for a one-sided *t* test comparing the +polyamine treatment to the negative-control treatment.

10.1128/mBio.01091-21.7 FIG S2 Loci for genes involved in polyamine metabolism (marked with a *) in the two strains of SAR11 under study, HTCC1062 and HTCC7211. Gene abbreviations and corresponding enzyme names are listed here; gene abbreviations in parentheses represent the homologous gene. *speB*, agmatinase; *ppiA*, peptidylprolyl cis-trans isomerase precursor; *fadB*, 3-hydroxyacyl-CoA dehydrogenase; *potC*, spermidine/putrescine ABC transporter, permease; *potB*, permease; *potD*, SBP; *potA*, ATP-binding protein; *prr*, betaine-aldehyde dehydrogenase; *gab*, glutarate 2-hydroxylase; *oocM*, octopine transporter, permease; *occQ*, octopine transporter, permease; *occt*, octopine transporter, SBP; *speE*, spermidine/spermine synthase; *pfs* (*mtnN*), 5′-methylthioadenosine/*S*-adenosylhomocysteine nucleosidase; *apt*, adenine phosphoribosyltransferase; *mtnA*, methylthioribose-1-phosphate isomerase; *mtnP*, methylthioadenosine phosphorylase; *speC*, lysine/ornithine decarboxylase; *dys2*, deoxyhypusine synthase-like protein; *speB1*, arginase; *yhhQ*, uncharacterized ACR; *rpmB*, 50S ribosomal protein L28; *pyrD*, dihydroorotate dehydrogenase; *lysS*, lysine-tRNA ligase; *aldH* (kauB), 4-aminobutyraldehyde dehydrogenase; *bioA* (*spuC*), putrescine-pyruvate aminotransferase; *ald*, alanine dehydrogenase; *argD* (*gabT*), acetylornithine aminotransferase; *argF*, ornithine carbamoyltransferase; *cycM*, cytochrome C; *kdsB*, 3-deoxy-manno-octulosonate cytidylyltransferase; *gabD*, succinate-semialdehyde dehydrogenase. Download FIG S2, PDF file, 0.09 MB. Copyright © 2021 Noell et al. 2021 Noell et al. https://creativecommons.org/licenses/by/4.0/ This content is distributed under the terms of the Creative Commons Attribution 4.0 International license.

In the extracellular fractions of both strains, there were no significant differences between the experimental treatment and the no-cell control (polyamines added, no cells), except for AGM in HTCC1062 (*P* value of 0.04, one-sided *t* test) (Fig. 2B and D). In HTCC1062, all five compounds were lower in concentration in the experimental treatment than in the no-cell control (Fig. 2B). For HTCC7211, all five compounds were at similar concentrations between the two treatments, except for AGM, which was lower in the experimental treatment (*P* value of 0.06, one-sided *t* test).

### Flow cytometry experiment.

**FIG 3:**
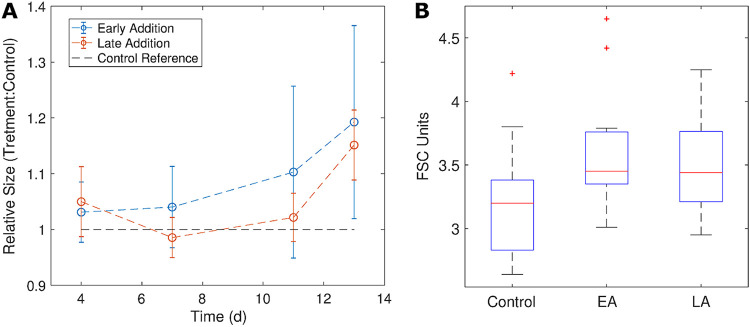
Changes in cell size in “*Candidatus* Pelagibacter” strain HTCC7211, approximated by forward scatter (FSC), were monitored using flow cytometry in response to the addition of 250 nM polyamines either at the beginning of the experiment (early addition) or after 4 days of growth (late addition). The first measured time point was 4 h after polyamines were added to the late addition cultures. (A) Mean FSC of the population at each time point from experimental treatments after normalization to the mean of the control treatment cultures (no polyamines added). Error bars are the standard deviation of quadruplicate replicates. (B) Boxplots of the mean FSC values for all time points of the three treatments. The red line is the median value, and the bottom and top edges of the box indicate the 25th and 75th percentiles. Statistical outliers (red +) are more than 1.5 times the interquartile range away from the bottom or top of the box.

Based on the very high intracellular polyamine concentrations measured in HTCC7211 cells in experimental treatments, we postulated that cells would change in size to accommodate the influx of polyamines. To test this, we used flow cytometry to monitor the forward scatter (FSC), a proxy for cell size, of nutrient-replete HTCC7211 cultures exposed to either no polyamines (control) or 250 nM each polyamine added at the beginning of growth (early addition) or after 4 days of growth (late addition). On average, both experimental treatments had higher FSC than the control (Fig. 3B). The mean FSC of the early addition cultures was consistently higher than that of the control across all measured time points (Fig. 3B). The mean FSC of the late addition cultures was similar to that of the early addition cultures 4 h after addition of polyamines to late addition cultures; it then decreased to control levels at day 7, finally increasing above the control level after day 11 (Fig. 3A).

### Carbon substitution experiments.

Growth experiments were used to examine whether the five polyamine compounds could substitute for two unusual growth requirements of SAR11 cells—pyruvate, or related compounds which lead to a branch of SAR11 metabolism that includes the biosynthesis of alanine, and glycine or related compounds, required for another branch of SAR11 metabolism that includes glycine synthesis. The five polyamine compounds were added together at final concentrations of 250 nM each as a replacement for either pyruvate or glycine, and the growth of the cultures was compared to that of the negative-control treatments with either no pyruvate or no glycine added. With both strains, experimental treatments with added polyamines achieved higher maximum cell densities and higher growth rates than negative controls, but the differences were not significant, indicating that these compounds did not fully substitute for glycine or pyruvate (Table 3).

**TABLE 3:** Growth rates and maximum cell densities for growth experiments with SAR11 cultures to determine if polyamines could substitute for pyruvate or glycine in their growth^*a*^

SAR11 strain	Treatment	Polyamines added?	Pyruvate added?	Glycine added?	Growth rate (day^−1^) ± SD	Max. cell density (cells/ml) ± SD
HTCC1062	Positive control	No	Yes	Yes	0.33 ± 0.02	3.27E7 ± 5.72E6
	Negative control	No	Yes	No	0.21 ± 0.003	4.19E6 ± 7.63E5
	Exptl	Yes	Yes	No	0.23 ± 0.02	5.30E6 ± 1.66E6
	Negative control	No	No	Yes	0.14 ± 0.03	1.25E6 ± 4.65E5
	Exptl	Yes	No	Yes	0.15 ± 0.01	1.36E6 ± 2.22E5
HTCC7211	Positive control	No	Yes	Yes	0.55 ± 0.00	1.48E8 ± 1.98E7
	Negative control	No	Yes	No	−0.11 ± 0.01	6.64E4 ± 1.17E3
	Exptl	Yes	Yes	No	−0.12 ± 0.1	7.57E4 ± 2.37E4
	Negative control	No	No	Yes	0.19 ± 0.02	1.04E6 ± 2.55E5
	Exptl	Yes	No	Yes	0.22 ± 0.03	1.76E6 ± 3.41E5

a All five polyamine compounds were added at a 250 nM final concentration each in place of either pyruvate or glycine and compared to negative controls with either no pyruvate or glycine added. Pyruvate was added at 100 μM and glycine at 50 μM; 10 μM methionine and vitamins were added to all cultures in artificial seawater medium (see Materials and Methods).

### Nitrogen substitution growth experiments.

**FIG 4:**
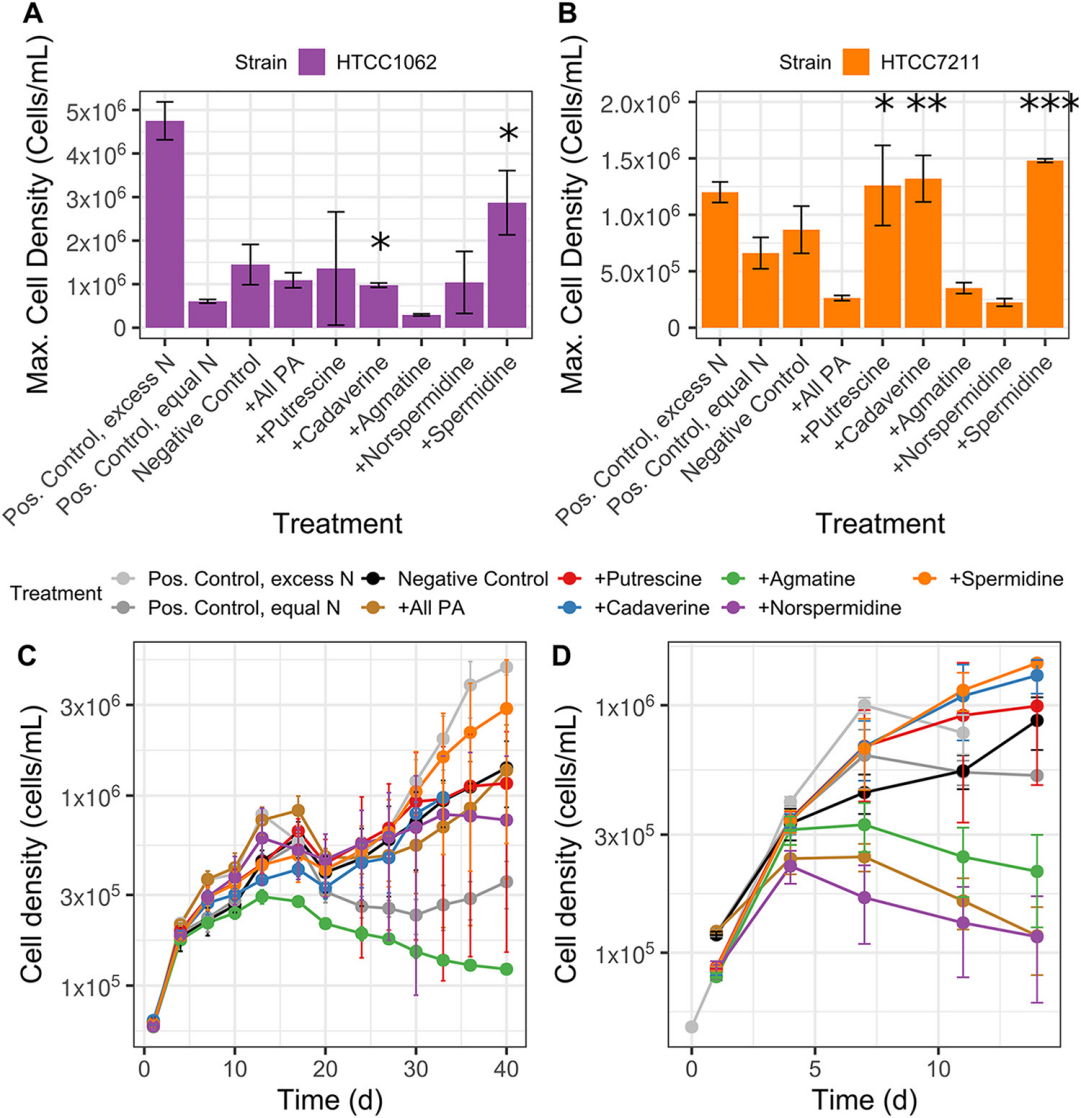
Several polyamine compounds are used by SAR11 cells as a nitrogen (N) source, as indicated by higher maximum cell densities (A and B) during growth experiments compared to control cultures. (C and D) Growth curves of cultures, from which the maximum cell densities were calculated. SAR11 cells, either HTCC1062 (A and C) or HTCC7211 (B and D) were grown on a modified ASM recipe (see Materials and Methods) without any N source. Negative-control cultures had no N added. Positive-control cultures were grown with N in the form of (NH_4_)_2_SO4, with either 400 μM (pos. control, excess N) or with equimolar N equal to the amount of N (245 nM N) added in the polyamine treatments (pos. control, equal N). Experimental treatments had equal amounts of N (245 nM N) added in the form of either all five polyamine compounds together, the final concentration of 150 nM for each compound (+all polyamines), or with individual polyamine compounds. Cultures were started from cultures that had been grown to the late exponential phase on the same media without any N, to eliminate any carryover from previous growth. Error bars represent the standard deviation of triplicate samples. *, significantly higher maximum cell density compared to the pos. control, equal N treatment (one-sided *t* test, *P* < 0.05); **, significantly higher maximum cell density compared to both pos. control, equal N and negative-control treatments (one-sided *t* test, *P* < 0.05); ***, significantly higher maximum cell density compared to all three control treatments (one-sided *t* test, *P* < 0.05). For HTCC7211, results from two separate experiments are shown together.

Additional growth experiments were used to determine if polyamine compounds could serve as nitrogen (N) sources. To eliminate organic sources of N in the media, glycine (required by SAR11 cells) and methionine (required as a sulfur source) were replaced with oxaloacetate and dimethylsulfoniopropionate (DMSP) in a modified artificial seawater medium (27). These substitutions resulted in ∼100-fold lower maximum cell densities due to oxaloacetate being a weak glycine substitute. The five polyamine compounds were added to SAR11 cultures grown in the modified artificial seawater (ASW) medium either all together at a final concentration of 150 nM for each compound, or individually at concentrations that provided equivalent amounts of N (245 nM N). Maximum cell densities were compared to a negative control with no added N (–C, –N), a positive control with excess N in the form of ammonium sulfate (+C, excess N), and a positive control with equimolar N in the form of ammonium sulfate (+C, equimolar N) (Fig. 4).

For HTCC1062, addition of two compounds, CAD and SPD, resulted in significantly higher maximum cell densities than the equimolar positive control (maximum cell densities and *P* values in Table S2) (Fig. 4). Addition of NSD, PUT, and all polyamines combined resulted in higher, albeit nonsignificant, maximum cell densities than for the equimolar positive control. Only addition of SPD resulted in a higher cell density than the negative control, but the difference was not significant. For HTCC7211, addition of SPD resulted in a significantly higher maximum cell density than those in all three controls. Cultures to which CAD was added had significantly higher maximum cell densities than the equimolar control and the negative control. Cultures with PUT added had a significantly higher maximum cell density than only the equimolar control. In both strains, the addition of AGM resulted in lower maximum cell densities than those of any control treatments; in HTCC7211, the treatments with NSD and with all polyamines combined also had lower maximum cell densities than those of any controls. Interestingly, diauxic growth was observed in HTCC1062, with an early peak around 14 days and a larger peak later around 40 days (Fig. 4), which was not observed in HTCC7211.

10.1128/mBio.01091-21.2 TABLE S2 Maximum cell densities for SAR11 cultures grown with polyamine compounds as the sole nitrogen source with corresponding *P* values. All *P* values are from a one-sided *t* test to assess whether the treatment had a significantly higher maximum cell density than the corresponding control. When an experimental treatment’s maximum cell density was lower than or equal to the corresponding control, a *t* test was not conducted. Download Table S2, DOCX file, 0.02 MB. Copyright © 2021 Noell et al. 2021 Noell et al. https://creativecommons.org/licenses/by/4.0/ This content is distributed under the terms of the Creative Commons Attribution 4.0 International license.

### Metabolic pathways.

Figure 1 shows genes for polyamine metabolism for the two SAR11 strains used in this study, overlaid on common pathways for polyamine metabolism (8, 12, 33, 38–40). In both strains, AGM is postulated to be converted by agmatinase (*speB*) to PUT, which is catabolized by the transamination pathway, since neither strain encodes the final enzyme in the γ-glutamylation pathway, and the transamination pathway was upregulated in SAR11 cells in response to PUT addition (28). CAD is likely metabolized to succinate via the lysine degradation pathway. Many genes involved in polyamine metabolism are known to be multifunctional (in terms of broad catalytic/substrate range) in other cell types (33, 41), as we also predict in SAR11 (Fig. 1; Fig. S2).

In metabolic reconstruction from genome sequences (42), we found that neither SAR11 strain encoded a clear pathway of SPD or NSD metabolism, although both compounds were taken up from the medium and metabolized (Fig. 2 and 4). In our analysis of genomes, we found that both strains lack the genes present in several *Vibrio* strains (43) that are responsible for producing and metabolizing NSD via carboxynorspermidine. Furthermore, we found that neither SAR11 strain has homologs for the canonical genes responsible for SPD metabolism—SPD dehydrogenase (s*pdH*), which cleaves SPD to produce 1,3-diaminopropane (DAP) and 4-aminobutanal, and SPD acetyltransferase, which converts SPD to acetylspermidine, a less toxic version of SPD. To explain the uptake and metabolism of SPD and NSD despite the lack of canonical metabolic genes for these compounds, we hypothesized that either the SPD synthase enzyme, SpeE, is bi-directional, producing PUT from SPD, or there is another, unknown enzyme capable of cleaving SPD. SpeE is not known to be bi-directional in other bacteria (44, 45). A possible candidate enzyme for SPD metabolism was discovered in both strains of SAR11 during metabolic reconstruction, *dys2*, a putative deoxyhypusine synthase (*dhs*) gene (Fig. S2). In other bacteria, the enzyme produced by *dhs* usually acts as a homospermidine (HSD) synthase, a promiscuous enzyme capable of acting on multiple polyamines in addition to its native function of producing HSD from PUT (46–48).

**FIG 5:**
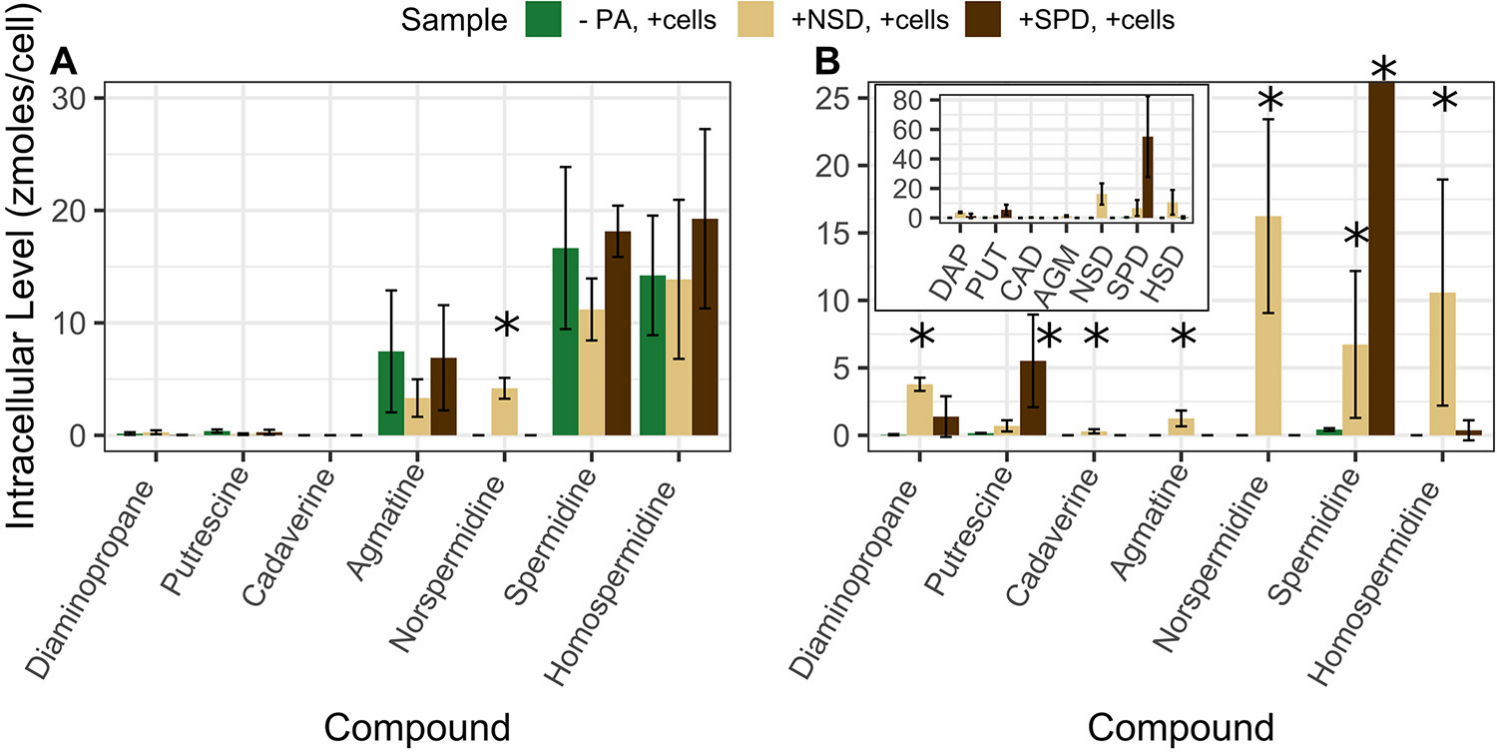
(A and B) Intracellular polyamine levels indicating pathways for norspermidine (NSD) and spermidine (SPD) metabolism in (A) HTCC1062 and (B) HTCC7211. Cultures were grown in nutrient-replete conditions either without any added polyamines, with 500 nM NSD, or with 500 nM SPD added. Cells were harvested at the late exponential phase, and intracellular polyamines were extracted as described in Materials and Methods. * indicates a significantly higher level of that compound compared to the control treatment with no polyamines added (one-sided *t* test, *P* < 0.05). Error bars are the standard deviation of quadruplicate cultures.

To test our hypothesis and differentiate between these two alternative routes of SPD metabolism and identify pathways of NSD metabolism in SAR11, we used fingerprinting to search for possible by-products of SPD and NSD metabolism, including DAP and HSD. If SAR11 cells use the reverse SPD synthase reaction to metabolize SPD, the products would be PUT and *S*-adenosylmethionine, while the by-products of the Dys2 enzyme, if it is an HSD synthase, would primarily be HSD and DAP. We compared polyamine levels in SAR11 cells grown either without any polyamines or with either 500 nM SPD or NSD (Fig. 5). As expected, in both strains, the treatments with SPD or NSD added had higher levels of that compound in the respective treatment (one-sided *t* test, *P* values of 0.02 and 0.003 for +SPD and +NSD versus the negative control, respectively; *P* values were 0.06 and 0.2 for HTCC1062), indicating an uptake of those two compounds by both strains.

In HTCC7211, the +SPD cultures also had significantly higher levels of PUT than the negative control (one-sided *t* test, *P* value of 0.05) but only a slight, nonsignificant increase in DAP (Fig. 5B). In the +NSD cultures, there were significantly higher levels of DAP than in the negative control (one-sided *t* test, *P* value of 0.001). There was also a significant increase in SPD and HSD (one-sided *t* test, *P* values of 0.05 and 0.04) in +NSD cultures compared to the negative control. In HTCC1062, there were no differences in the levels of any other compounds in the +NSD or +SPD treatments compared to those of the negative control, aside from SPD and NSD themselves and a nonsignificant increase in DAP in the +NSD treatment (Fig. 5A). The increase in DAP in HTCC7211 in both the +SPD and +NSD cultures and low levels of HSD in these treatments lend support to SpeE being responsible for SPD and NSD metabolism.

## DISCUSSION

SAR11 cells devote much of their cellular structure and energy to transport functions to compete for resources in the world’s nutrient-limited oceans (17, 18, 20). One of the primary transporters expressed by SAR11 cells, PotABCD, transports polyamines, yet little is known about the types and amounts of polyamines used by SAR11, the role of polyamines in SAR11 growth, and the pathways used by SAR11 for polyamine metabolism. Here, we show that SAR11 cells took up and metabolized all five polyamine compounds tested and concentrated polyamines to μM and even mM intracellular concentrations. Cells increased in size while concentrating polyamines, probably as a consequence of osmosis and increasing turgor pressure inside the cells. We also show that SAR11 cells primarily use polyamines as a N source and propose metabolic pathways for SPD and NSD, two compounds for which catabolic pathways in marine bacteria are uncertain.

The growth of SAR11 cultures was found to be inhibited by high polyamine concentrations (Fig. S1; Fig. 3). Polyamines are known to be toxic to bacteria when added to media at high concentrations (generally mM range), but the mechanism is not known (40, 49, 50). SAR11 cells often lack transcriptional regulation for carbon oxidation functions (15), and previous work indicated they do not upregulate metabolic enzymes for polyamines when N limited (27). The growth inhibition observed at high polyamine concentrations might be due to adverse effects of the buildup of polyamine compounds inside the cells. Similar results previously have been observed in cells experiencing metabolic pathway saturation (51). Intracellular concentrations of polyamines were higher in HTCC7211 than in HTCC1062, and HTCC7211 was also more susceptible to growth inhibition by polyamines, indicating that the buildup of polyamines inside the cells might be linked to growth inhibition (Fig. S1A and B; Table 2).

### Uptake of polyamine compounds by SAR11 cells.

Organisms produce intracellular polyamines for a variety of cellular processes, including stabilization of DNA, RNA, and proteins (3). The native polyamines produced by these strains (SPD and HSD in HTCC1062 and SPD alone in HTCC7211 [Fig. 2 and 5]) are consistent with past reports that alphaproteobacteria primarily make PUT, SPD, or HSD as polyamines (52). AGM and PUT, intermediates in the synthesis of SPD and HSD (Fig. 1), were also detected at low levels. The concentrations of native polyamines measured in SAR11 cells are similar to those measured in various phytoplankton (4) but ∼10-fold lower than those found in other alphaproteobacteria (52).

Both strains took up all five polyamines in excess of metabolic rates, causing higher (10- to 1,000-fold higher) intracellular concentrations in experimental treatments relative to negative controls (Fig. 2A and C). Intracellular levels were in the zeptomole (10^−21 mol^, or 1^3^ molecules)/cell range, similar to levels measured before in SAR11 cells for metabolites such as glycine betaine and DMSP (20, 53). Intracellular polyamines reached μM concentrations in HTCC1062 and mM concentrations in HTCC7211, 20 mM in the case of SPD in HTCC7211 (Table 2). The polyamine concentrations taken up by HTCC1062, although in the low μM range for some compounds, are likely still high enough to be used effectively in metabolism, given the low μM *K_m_* values measured for enzymes involved in polyamine metabolism in other cells (44, 54–57). In response to the large influx of polyamine compounds, HTCC7211 cells increased in size within hours, as indicated by the high level of FSC in the late addition treatment cultures compared to that of the negative-control treatment cultures at the first measured time point, taken 4 h after polyamines were added, with cell size increasing the most after several days of exposure (Fig. 3).

Substrate uptake in excess of metabolic rate has been observed previously in SAR11 cells with the osmolyte dimethylsulfoniopropionate (DMSP) (53). A parallel phenomenon, termed “luxury uptake,” has been described in phytoplankton that take up phosphorous and nitrogen in excess of their requirements and store them in organic forms for later use (58, 59). However, the excess substrate uptake we observed does not fit the canonical definition of luxury uptake, since the amount of N from the stored polyamines in SAR11 cells was far less than their requirement for a cell division; concentrated polyamines inside SAR11 cells in the experimental treatment made up only 0.005% for HTCC1062 and 0.27% for HTCC7211 of the cellular N quota, estimated at 0.11 fmol N/cell (37).

Theoretically, excess uptake such as what we observed enhances the ability of cells to exploit nutrient patches, giving them a cache of nutrients to process subsequently after exiting a nutrient patch. Moreover, given that SAR11 cells are by far the most abundant cell type in the ocean, luxury uptake by SAR11 cells could be a population-level strategy that lowers ambient nutrient concentrations to levels where SAR11 cells are more competitive for transport, effectively taking nutrients off the table. Past theoretical work has shown that superior competitors in patchy environments lower average nutrient concentrations (60). Our findings may stimulate further research aimed at understanding whether these cellular behaviors apply to other substrate compounds used by SAR11 cells, whether similar behavior is exhibited by other oligotrophs, and whether the imbalance between transport and metabolism we observed occurs in natural populations and plays an adaptive role by allowing transient storage of exometabolites inside cells.

Although HTCC1062 and HTCC7211 transported all five compounds from the media, there were few observable depletions of extracellular concentrations for any polyamines with either strain because the intracellular polyamine pools were small relative to the surrounding volume (Fig. 2C and D). The observed accumulation of intracellular polyamines was estimated to result in pmolar drawdowns of the dissolved polyamine pool, which in most cases was less than the precision of our measurements (Table S3). An exception was the accumulation of 239 pmol of intracellular SPD in HTCC7211, which should have produced a measurable depletion of SPD in the medium, yet no significant reduction was observed (standard deviation of 83.3 pmol). This observation suggests that HTCC7211 cells used other transported polyamines to synthesize SPD. To support this interpretation, HTCC7211 cells given only SPD had 10-fold lower SPD levels than when given all five compounds (compare Fig. 2C and Fig. 5B). It appears that transported polyamines are converted intracellularly to SPD, which then accumulates in HTCC7211.

10.1128/mBio.01091-21.3 TABLE S3 Intracellular polyamine measurements from fingerprinting experiments (see Fig. 2) fall within the error range of corresponding extracellular footprint measurements from the spent culture media. The intracellular polyamine levels in cells grown on polyamines were back-calculated to molar values based on the number of cells in the culture when harvested. The standard deviation (stdev) for the extracellular measurements of polyamine compounds from the corresponding spent media were also back-calculated to molar values for comparison. The only compound that clearly falls outside the error measurements is spermidine in HTCC7211. Download Table S3, DOCX file, 0.02 MB. Copyright © 2021 Noell et al. 2021 Noell et al. https://creativecommons.org/licenses/by/4.0/ This content is distributed under the terms of the Creative Commons Attribution 4.0 International license.

Our analysis suggests the PotABCD transport system in SAR11 is responsible for transporting the five polyamines we tested, given the structural similarity between these compounds and the absence of other candidates for polyamine transport functions. However, this remains to be experimentally validated, as was done previously for the glycine betaine transporter in SAR11, which was found to transport seven different substrates (20). Both strains of SAR11 lack homologs for CAD, NSD, or AGM transporters found in other bacteria. In Vibrio cholerae, NSD is transported by a *potABCD* homolog (61), but CAD and AGM have not previously been identified as substrates for *potABCD*. In Escherichia coli, PotABCD is primarily a PUT/SPD/SPM transporter (62), which could help explain the higher accumulation of these compounds in SAR11 cells, even when accounting for native PUT and SPD production. It is likely that other ABC transporters in SAR11 cells are also multifunctional (e.g., able to transport a wider range of substrates than canonically), given the use of a wide variety of amino acids and carboxylic acids by SAR11 cells (63–65).

Interestingly, there was a large difference between the two SAR11 strains in the amounts of polyamines taken up. Polyamine concentrations in HTCC7211 were 40- to 500-fold higher than those of HTCC1062, despite HTCC7211 being exposed to 2-fold lower concentrations of polyamines. This difference cannot be explained entirely by cell size; HTCC7211 cells are only ∼1.3-fold larger, as measured by C content, than HTCC1062 cells (37). The differential could be because the HTCC7211 transport system has a higher *V*_max_ for polyamine transport than HTCC1062, due either to the properties of the proteins themselves (one of the two permease proteins involved in polyamine transport, PotB, is only 82% identical), differing abundances of transport proteins, or the cytoarchitecture of the cells. There were no major differences between the two strains in the presence/absence of polyamine metabolic genes, nor in the location of those genes (Fig. S2). One possible ecological explanation for the difference between these two strains, if they are typical of the ecotypes they represent, is that HTCC1062, a member of the primarily coastal subclade of SAR11, may have been influenced by selection that limits toxic buildups of polyamines at the higher polyamine concentrations found in coastal regions. HTCC7211, a member of a primarily open ocean subclade of SAR11, would rarely experience the high polyamine concentrations found in coastal regions and so might not experience selection to limit intracellular buildups.

### Use of polyamines by SAR11 cells.

SAR11 cells have unique growth requirements, needing a reduced sulfur source (e.g., methionine or methane thiol), a glycine source, specific vitamins, and a carbon source that can serve as a precursor to alanine (usually pyruvate) (64). Most of the tested polyamine compounds are predicted to be metabolized to succinate, a tricarboxylic acid (TCA) cycle intermediate (Fig. 1). In previous work, succinate did not substitute for pyruvate in HTCC1062, in accord with our experimental findings (66). This does not rule out the use of polyamines as an energy source, however. Other small compounds have been found previously to be used by SAR11 cells only as an energy source and not as a pyruvate substitute (20, 65). Polyamines are also required for a variety of other cellular processes, and it is likely that SAR11 cells used the supplied polyamines in those processes in addition to metabolizing them.

Both strains of SAR11 tested could use several polyamine compounds (SPD, CAD, and PUT) as a N source, with SPD supporting the highest maximum cell density of any of the polyamines (Fig. 4A). NSD does not appear to be a N substitute for either strain of SAR11 at the NSD concentration tested, but it is transported (Fig. 5). The use of multiple polyamines as N sources is consistent with previous reports that SAR11 cells use a variety of organic N-containing compounds as N sources (27).

Interestingly, two compounds (AGM and NSD) were inhibitory to SAR11 growth under N-limiting conditions (Fig. 4). One potential cause for the AGM inhibition is the by-product of AGM degradation by the agmatinase enzyme, urea. Neither SAR11 strain encodes a urease (67). We speculate that the influx of AGM causes a build-up of inhibitory urea in cells, a process that is known to occur in oligotrophs due to metabolic pathway saturation (15, 51). This does not, however, preclude AGM from also being used as a N source by the cells at environmental AGM concentrations.

Surprisingly, all HTCC1062 cultures, including controls, exhibited diauxic growth during N-substitution experiments, while HTCC7211 cultures, under similar conditions, did not. Diauxic growth is generally observed when cells switch from using one source of nutrients to another. However, the cultures used to start these experiments were acclimated to the same medium (without N) prior to the experiment starting. Previously, diauxic growth was observed in HTCC7211 grown on alternate P sources, which was attributed to the switch from using inorganic P to organic P sources (68). Another unexpected observation, found in both strains and across several repetitions, was that the equimolar positive control always had a lower maximum cell density than the negative control with no N added.

### Metabolic pathways for spermidine and norspermidine.

SPD metabolism has been observed in marine bacteria without a spermidine dehydrogenase gene (*spdH*) (9, 11, 28). It has been speculated that the enzyme that synthesizes SPD from PUT, SpeE, is bi-directional, although this activity was not confirmed experimentally (9), and SpeE is known to not be bi-directional in other cell types (44, 45). In HTCC7211, it appears that SPD is primarily metabolized via the reverse SPD synthase reaction, not via the Dys2 enzyme, as no significant increase in HSD was detected, while an increase in PUT was observed (Fig. 5B). The SpeE enzyme in SAR11 previously has been found to be multifunctional, both in terms of being a multidomain protein as the result of a gene fusion event and showing broad substrate range, with high biosynthetic activity on multiple polyamines (69). Our data suggest that this enzyme is multifunctional not only in its substrate range, but also in its ability to carry out catalytic reactions in reverse of its usual biosynthetic activities, which is known to not occur in other cell types (44). The results from HTCC1062 on SPD metabolism are not as clear, as no other differences between the +SPD treatment and the negative control were observed aside from an increase in SPD (Fig. 5A).

We propose that NSD is metabolized in HTCC7211 by the enzyme SpeE, similar to SPD, since we observed increased levels of DAP in the NSD treatment, and the SpeE enzyme in SAR11 has a wide substrate rage (Fig. 5B). With these data, we cannot rule out the Dys2 enzyme metabolizing NSD. In HTCC7211, there was also an increase in SPD and HSD in the +NSD treatment (Fig. 5B). The Dys2 enzyme in SAR11 cells may be responsible for producing SPD and HSD from the excess NSD and DAP, since *dhs* homologs in bacteria are known to produce SPD from PUT and DAP, in addition to producing HSD from PUT (46, 47, 70). It appears that the *dys2* gene in both SAR11 strains is not primarily acting as an HSD synthase, since there was relatively low production of HSD under any condition, in contrast to other prokaryotes with an HSD synthase gene where HSD is the sole polyamine present (47, 70).

We evaluated the hypothesis that SPD synthase might be catalyzing reactions that are the reverse of its ordinary action of synthesizing polyamines. We explored thermodynamic models that predicted the energies of the compounds in the primary reaction catalyzed by SPD synthase (forming SPD from PUT; Fig. S3), without considering entropy terms (Text S1; Table S4). The estimated ΔE value for the total reaction was positive (14.32 kcal/mol) when water was used as the proton acceptor (it is expected that ΔH° values will be quite similar to ΔE values) (Table S5). More favorable acceptors (e.g., imidazole) easily yield negative ΔE values of −23.49 kcal/mol (Table S5). Our findings suggest that the direction of this reaction is easily tunable by including proton carriers of various strengths. This calculation treated each chemical species as an isolated unit and did not take any account of intermolecular interactions. We did not consider the very high intracellular concentrations of polyamines we observed experimentally, which might further drive this reaction in the reverse of its canonical function in polyamine biosynthesis.

10.1128/mBio.01091-21.4 TABLE S4 Computed energies for the compounds of interest in the spermidine synthase reaction (Fig. S3). I, *S*-adenosyl-3-(methylsulfanyl)-propylamine; III, *S*-methyl-5′-thioadenosine. Download Table S4, DOCX file, 0.01 MB. Copyright © 2021 Noell et al. 2021 Noell et al. https://creativecommons.org/licenses/by/4.0/ This content is distributed under the terms of the Creative Commons Attribution 4.0 International license.

10.1128/mBio.01091-21.5 TABLE S5 Estimated energies of reaction for the canonical spermidine synthase reaction (Fig. S3) using either water or imidazole as the proton acceptor. It is expected that ΔH° values will be quite similar to ΔE values. Download Table S5, DOCX file, 0.01 MB. Copyright © 2021 Noell et al. 2021 Noell et al. https://creativecommons.org/licenses/by/4.0/ This content is distributed under the terms of the Creative Commons Attribution 4.0 International license.

10.1128/mBio.01091-21.8 FIG S3 Canonical reaction catalyzed by the spermidine synthase enzyme. B: is the base used in the thermodynamic calculations as the proton acceptor in the reaction. Download FIG S3, PDF file, 0.02 MB. Copyright © 2021 Noell et al. 2021 Noell et al. https://creativecommons.org/licenses/by/4.0/ This content is distributed under the terms of the Creative Commons Attribution 4.0 International license.

10.1128/mBio.01091-21.10 TEXT S1 Explanation and further description of the thermodynamic calculations conducted for the reaction catalyzed by spermidine synthase. Download Text S1, DOCX file, 0.02 MB. Copyright © 2021 Noell et al. 2021 Noell et al. https://creativecommons.org/licenses/by/4.0/ This content is distributed under the terms of the Creative Commons Attribution 4.0 International license.

### Conclusion.

Some properties of plankton cells that are important to understanding and modeling their behavior in natural ecosystems can only be measured by experimentation. Recently, we demonstrated very low whole-cell affinities and multifunctionality in the osmolyte transport system of cultured SAR11 cells. We attributed these competitively advantageous cell properties to synergism between kinetic features of the glycine betaine transporter ProXYZ and unusual aspects of SAR11 cell architecture, notably their small size and large periplasm packed with abundant substrate binding proteins (20). Here, we investigate SAR11 metabolism of polyamines, which are transported into cells by the highly abundant SAR11 transporter system PotABCD. We find that this system is also multifunctional and that the two SAR11 strains metabolized a variety of polyamines, which served the cells as N sources. Our growth experiments did not find that polyamines were able to substitute for glycine (or related compounds) or pyruvate (or related compounds) in their growth. In previous work, we have shown that several C1 compounds are used by SAR11 as energy sources via tetrahydrofolate-mediated oxidation but not as a source of carbon for biomass production (65), which may be the case with polyamines. Our data strongly support the hypothesis that SAR11 uses many polyamines via a simplified system of few enzymes and a single transporter. They mainly use these compounds as an N source and perhaps to supplement their intracellular polyamine pool, potentially important adaptations in N-limited marine systems.

Polyamine transport rates exceeded metabolic rates, leading to mM intracellular polyamine accumulations and an increase in cell size over a period of days. We propose that SAR11 cells use the multifunctional enzyme spermidine synthase, SpeE, in reversible reactions that can both produce SPD and catabolize SPD and NSD. The findings we report indicate enzyme multifunctionality expands the range of DOM compounds these cells harvest, which may partially explain how these cells attain high success in competition for DOM resources. Our findings also support previous observations which indicated SAR11 cells concentrate some metabolites during pulses of availability, metabolizing them subsequently (53). In principle, this cell behavior could increase the success of SAR11 cells in competition for nutrient patches, but further experimental work and modeling are needed to evaluate this hypothesis. In any case, the properties of cells that we uncovered here are neither typical nor trivial; they change our understanding of how competition for DOM resources has led to the emergence of specialized cell types and will likely inform future experimental research and modeling aimed at understanding cell evolution and the ocean carbon cycle.

## MATERIALS AND METHODS

### SAR11 growth and washing.

The protocol for SAR11 growth conditions, cell counts, and washing has been previously reported (20). Briefly, “*Candidatus* Pelagibacter” sp. HTCC7211 and “*Ca.* Pelagibacter ubique” HTCC1062 were grown in artificial seawater (ASW) amended with 100 μM pyruvate, 50 μM glycine, 10 μM methionine, and SAR11-specific vitamins at either 25 or 16°C (HTCC7211 and HTCC1062, respectively) in 12 h light/dark (64, 66). For testing polyamines as a nitrogen (N) source, a modified ASW was used without inorganic N (ammonium sulfate). This modified ASW was amended with 100 μM pyruvate, 50 μM oxaloacetate (instead of glycine), 1 μM dimethylsulfoniopropionate (DMSP; instead of methionine), and vitamins, since both glycine and methionine provide potential organic N sources (27).

In the growth experiments testing polyamines as a N source, there were two positive controls, a N-replete positive control, with 400 μM ammonium sulfate added, and an equimolar N-positive control, containing the same stoichiometric ammonium sulfate concentration as the experimental cultures with organic N sources (polyamines). For growth experiments, cell counts were taken at least twice a week, and growth rates and maximum cell densities were calculated as described previously (64). For growth experiments, a starting cell density of 1E5 cells/ml was used; a density of 5E4 cells/ml was used in experiments using the modified ASW, as cells grew to a lower maximum cell density in this medium. For the experiments testing polyamines as a N source, cultures were started from cultures grown without N to late exponential phase to exhaust any carryover N.

For footprinting/fingerprinting experiments, cells were harvested using centrifugation (Beckman-Coulter J2-21) at 10°C at 30,000 × *g* for 90 min. Cell pellets were resuspended in Tris-EDTA (TE) buffer and pelleted again (Beckman-Coulter Ultracentrifuge) at 12°C at 48,000 × *g* for 60 min (20).

### Footprinting and fingerprinting experiments.

To measure the identity and quantity of polyamine compounds used by SAR11 cells, a panel of five common polyamine compounds was added to SAR11 cultures at the beginning of growth under normal growth conditions. The polyamine compounds used were putrescine (PUT), cadaverine (CAD), agmatine (AGM), norspermidine (NSD), and spermidine (SPD). Each polyamine compound was added at a final concentration of 500 nM for HTCC1062 or 250 nM for HTCC7211; 250 nM was used for HTCC7211 as they were found to be inhibited in growth at 500 nM (Fig. S1A and B). Concentrations used were about at 100× ambient polyamine concentrations for several reasons—to allow for accurate quantification of polyamines, to match the high densities of cells used, which were also about 100× average cell densities in the ocean, and to match previous amendment studies with polyamines and other, similar compounds (28, 30, 71). A negative-control treatment with SAR11 cells but no polyamines added was used to measure native polyamine production and background polyamine concentrations in the ASW. A no-cell control with polyamines and no SAR11 cells was also included to account for degradation of polyamines, extraction efficiency during SPE, and carryover polyamines in the intracellular measurements. All treatments were done in quadruplicate. Samples were taken for cell counts twice a week; samples for extracellular (footprint) and intracellular (fingerprint) polyamine measurements were taken in the late exponential phase.

The metabolic pathways of two of the polyamine compounds, SPD and NSD, are not clear in SAR11 cells. Thus, further fingerprinting experiments were carried out with SPD and NSD added separately to cultures at 500 nM. Several possible metabolic intermediates and by-products were measured in these cells and compared to a negative control with no polyamines added.

### Intracellular polyamine extraction.

Intracellular polyamines were extracted from SAR11 cells using a method adapted from targeted marine metabolomics studies (72). Cells were pelleted and washed in TE buffer as described above. The supernatant was then completely removed, and the cell pellet was resuspended in 10 μl of fresh TE buffer. The volume was determined via an analytical balance. Then, 1 μl of resuspended cells was removed and diluted 1:200 in TE buffer to determine cellular abundance for per-cell normalization. Cells were lysed by adding 100 μl of cold methanol (MeOH; LC-MS grade) to the cell suspension, followed by the addition of 300 μl of cold 1 M acetic acid (72). Cell lysis and polyamine extraction were completed by shaking samples on high for 5 min, followed by rest in ice for 1 min to avoid overheating, repeated three times. A liquid-phase extraction was used to remove hydrophobic components of the cellular matrix; 400 μl of chloroform (LC-MS grade) was added, and the samples shaken for 1 min, followed by centrifugation for 5 min at 5,000 rpm to achieve phase separation. The aqueous layer containing the polyamines was transferred into a new tube, and the organic layer was discarded. The resulting sample was concentrated via drying under a nitrogen stream at 30°C. The dried samples were resuspended in 30 μl of 50:50 1 M acetic acid:acetonitrile (73), weighed, and analyzed as described below. When 125 nM standards of each compound were extracted using this method, minimal degradation was observed, with over 60% recovery (Table S1). Because no intracellular polyamine standard reference exists, the reported intracellular measurements in this paper were not corrected for recovery efficiency.

10.1128/mBio.01091-21.1 TABLE S1 Recoveries for each of the polyamine compounds using the two extraction methods used. Extracellular recovery is the recovery percentage of standards extracted from artificial seawater (ASW) media using solid-phase extraction (SPE); 500 nM standards of each compound were added to 10 ml ASW and extracted as described in Materials and Methods. The reported values are the average and standard deviation of triplicate samples. For the intracellular recovery, 125 nM standards of each compound in 10 μl volumes were extracted using the intracellular extraction method described in Materials and Methods. The reported values are the average and standard deviation of duplicate samples. Download Table S1, DOCX file, 0.02 MB. Copyright © 2021 Noell et al. 2021 Noell et al. https://creativecommons.org/licenses/by/4.0/ This content is distributed under the terms of the Creative Commons Attribution 4.0 International license.

### Extracellular SPE extraction.

Polyamines dissolved in the culture media were extracted using a solid-phase extraction (SPE) as described previously (73). A total of 10 ml of the supernatant from the initial centrifugation of cultures (described above) was used for each sample. Polyamines were extracted via gravity alone (nominal flow rate of 0.07 ml/min) onto a 1,000-mg Bond Elut-C_18_ SPE column (3 ml; Agilent) preconditioned with methanol and bicarbonate buffer at pH 12. Salts were removed from the column by washing three times with 1 ml of 0.1 M borate buffer, pH 12. Polyamines were eluted into cryovials with three washes of equal volumes of 1 M acetic acid and acetonitrile, final volume 5 ml. Greater than 85% recovery for CAD, PUT, NSD, and SPD was observed, while AGM had 48% recovery (Table S1), perhaps because of the high pKa of AGM (∼12) compared to that of the other four compounds (∼10). Artificial seawater (blanks) extracted using this method had only minimal concentrations (less than 4 nM) of NSD and SPD and none of the other three compounds (data not shown). Measured concentrations were not corrected for extraction recovery, so the values reported in this paper are conservative.

### LC-MS/MS analysis.

Quantification of polyamines was carried out using an Applied Biosystems 4000 Q-Trap triple quadrupole mass spectrometer with an electrospray ionization (ESI) interface, coupled to a Shimadzu LC-20AD liquid chromatograph (LC-MS/MS). The Applied Biosystems Analyst and ABSciex Multiquant software packages were used for instrument operation and quantification, respectively. A phenyl-3, 150 by 4.6-mm 5-μm high-pressure liquid chromatography (HPLC) column (GL Sciences) was used for chromatographic separations, using a 2.0-μm prefilter as a guard column (Optimize Technologies). The sample rack was cooled to 10°C to prevent degradation of polyamines. The column temperature was maintained at 40°C. HPLC mobile phases were MS grade water (Fisher) with 0.1% formic acid and MS grade acetonitrile (Fisher) with 0.1% formic acid. A 10-min binary gradient with a flow rate of 0.8 ml/min was used. The initial concentration of 3% acetonitrile ramped to 30% acetonitrile in 5 min. The column then reequilibrated at 3% acetonitrile for 5 min. The ESI source used a spray voltage of 5,200 V and a source temperature of 600°C. The sheath gas pressure was 50 lb/in^2^, and the auxiliary gas pressure was 40 lb/in^2^. The mass spectrometer was run in positive ion mode. Compound-specific multiple reaction monitoring (MRM) parameters, column retention times, and limits of detection (LOD) are presented in Table 1. The instrumental limits of detection (Table 1) were calculated as three times the standard deviation of six runs of the lowest detectable standard (5 nM). The sample injection volume was 10 μl, and samples were analyzed in triplicate. Samples and standards were all analyzed in a 50:50 acetonitrile:acetic acid mix. Samples were randomized prior to analysis. ^13^C-spermidine [spermidine-(butyl-^13^C4) trihydrochloride; Sigma-Aldrich] was added as an internal standard (IS) for quantification and to compensate for matrix effects. Compound concentrations that are listed as zero in figures and tables in this paper were below the LOD. LC-MS analysis was conducted at the Oregon State University Mass Spectrometry Core Facility. Data analysis was conducted in the R software environment (R Core Team, 2015), and all figures were created using the Ggplot2 software package for R (84, 85).

### Flow cytometry analysis.

Flow cytometry was used to monitor changes in cell size and morphology in HTCC7211 cultures in response to exposure to polyamines. Three treatments were used, all with cells grown under nutrient-replete conditions, with quadruplicate cultures for each treatment; cultures were started at a cell density of 5E4 cells/ml. The negative control had no polyamines added; in one experimental treatment (early addition), cultures were started with 250 nM each polyamine added to the media; in the other experimental treatment (late addition), 250 nM each polyamine was added after 4 days of growth, when they had passed 5E5 cells/ml density. Samples were taken twice a week until they reached early stationary phase. Cells were stained with SYBR green I prior to analysis on a Becton, Dickinson influx cell sorter (BD ICS). The BD ICS was equipped with a 488-nm laser and detectors for forward scatter (FSC), side scatter (SSC), and fluorescence at 530 nm, the emission wavelength of SYBR green I, and 692 nm. Prior to sample analysis, the instrument was aligned according to the manufacturer’s specifications. See references 74 and 75 for additional information on instrument specifications, alignment, and calibration procedures. Data were recorded for >50,000 cells per replicate per treatment. Flow cytometry files were analyzed using FlowJo (v.10.7.1), and cells were identified by their fluorescence in the 530-nm channel. The mean for each replicate for each detector channel was recorded and used in calculating the treatment means and standard deviations. Unfortunately, errors with the flow cytometer prevented observations of cells prior to day 4 of the experiment, and data are restricted to days 4, 7, 11, and 13. Cell concentrations determined on the flow cytometer are presented in Fig. S1G.

### Computational modeling.

The reaction catalyzed by spermidine synthase (Fig. S3) was computationally modeled to determine reaction energetics. Water and histidine were used separately as bases. Amine nitrogens in putrescine and spermidine were fully protonated, but the nitrogens in the adenosyl fragment were left unprotonated (neutral). First, conformational spaces for compounds I and III were explored using Spartan’14 (76), with the Merck molecular force field (MMFF) (Fig. S4). Density functional theory (DFT) studies were performed using Gaussian 16 (77). The B3LYP functional (78–81) was employed using the cc-pVDZ basis set (82). A self-consistent reaction field (SCRF) solvation model using water (83) was applied. All structures were optimized and showed only real vibrational frequencies. Self-consistent field (SCF) energies with solvation correction were used as the primary measure of molecular energy. Reaction energies were estimated using the minimum energy conformer for each compound.

10.1128/mBio.01091-21.9 FIG S4 Different conformations of the two more complex compounds in the spermidine synthase reaction. (A) “Compact” conformer of *S*-adenosyl-3-(methylsulfanyl)-propylamine. (B) “Extended” conformer of *S*-adenosyl-3-(methylsulfanyl)-propylamine. (C) “Compact” conformer of *S*-methyl-5′-thioadenosine. (D) “Extended” conformer of *S*-methyl-5′-thioadenosine. Download FIG S4, PDF file, 0.3 MB. Copyright © 2021 Noell et al. 2021 Noell et al. https://creativecommons.org/licenses/by/4.0/ This content is distributed under the terms of the Creative Commons Attribution 4.0 International license.

### Data availability.

The metabolomics data have been deposited in the EMBL-EBI MetaboLights database (doi: 10.1093/nar/gkz1019, PMID: 31691833) with the identifier MTBLS3146. The complete data set can be accessed at https://www.ebi.ac.uk/metabolights/MTBLS3146.
