# Supplementary figures and images for: SAR11 Cells Rely on Enzyme Multifunctionality To Metabolize a Range of Polyamine Compounds
Source: mBio. 2021 Aug 24;12(4):e01091-21. doi: 10.1128/mBio.01091-21 (PMC8437039; doi:10.1128/mBio.01091-21)

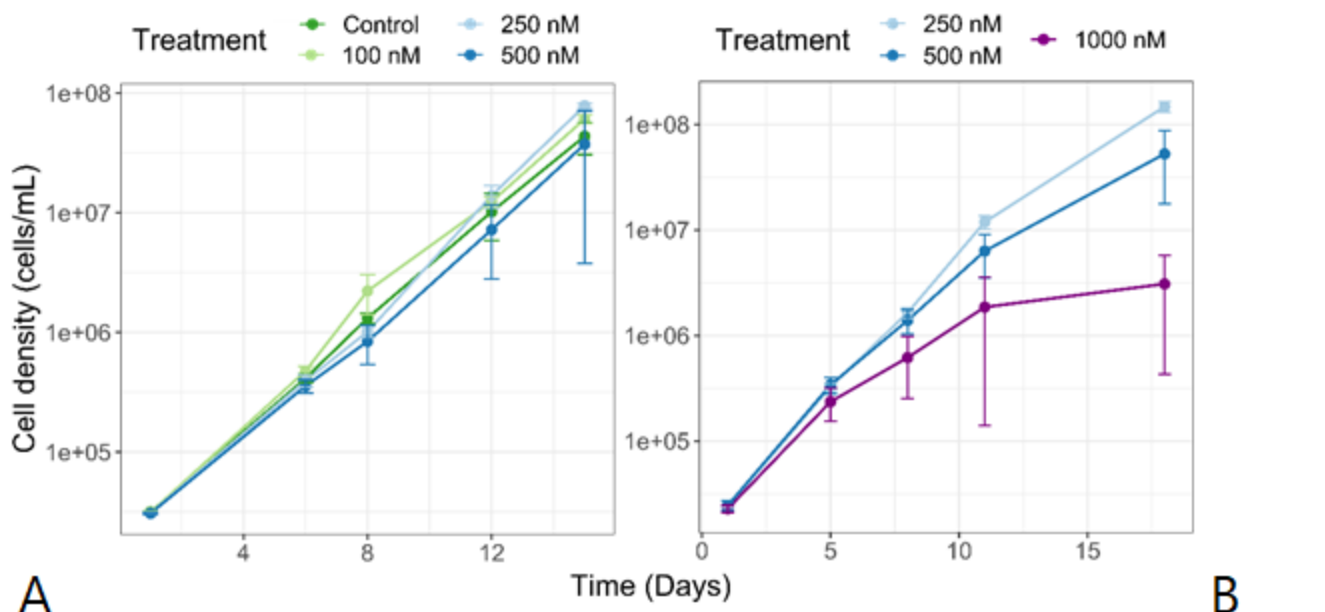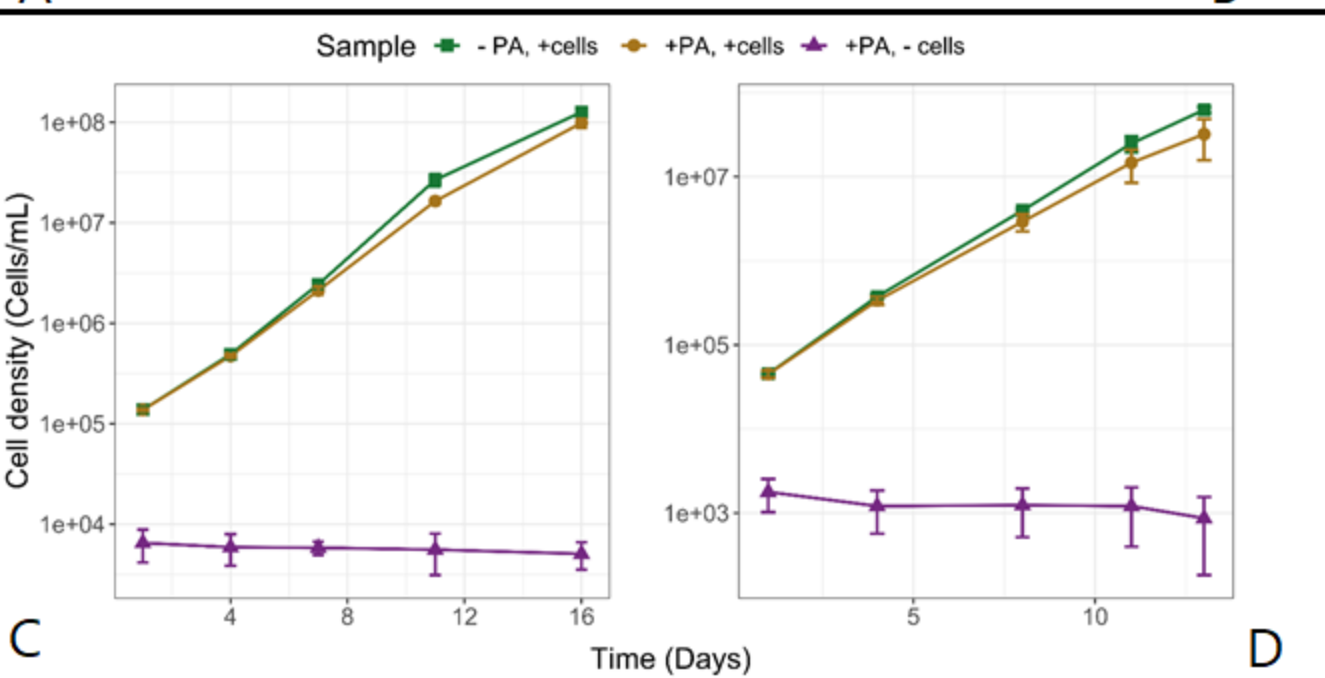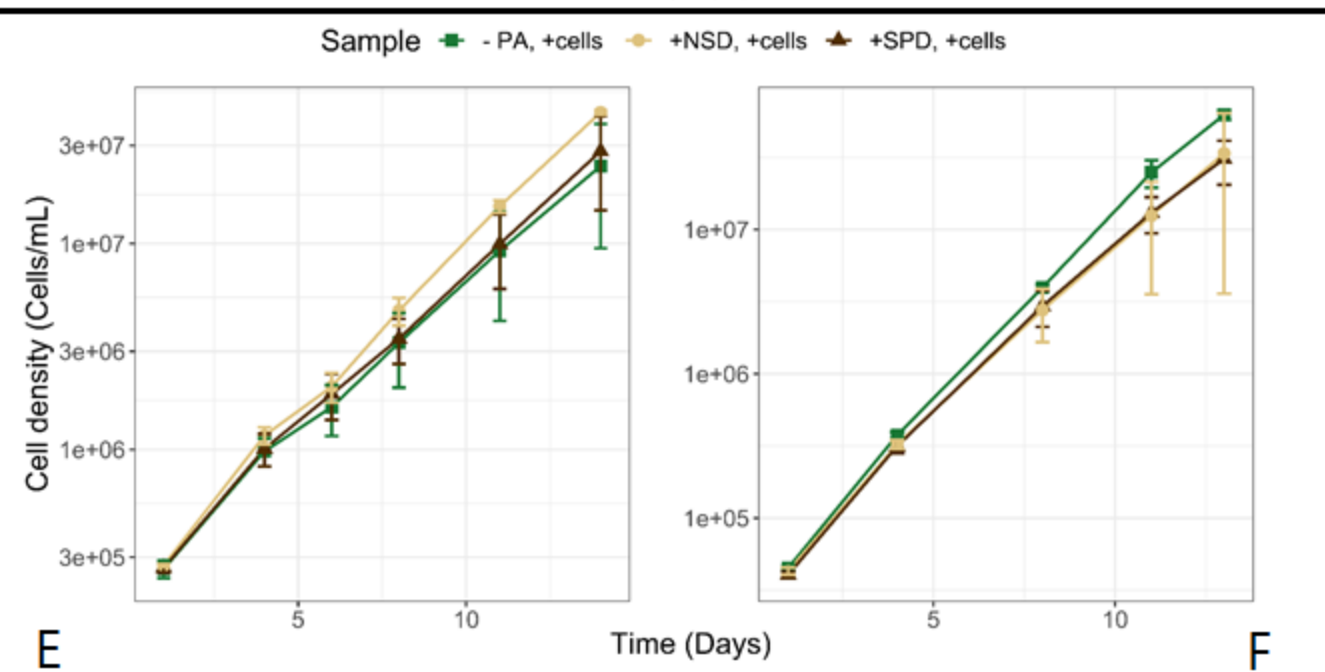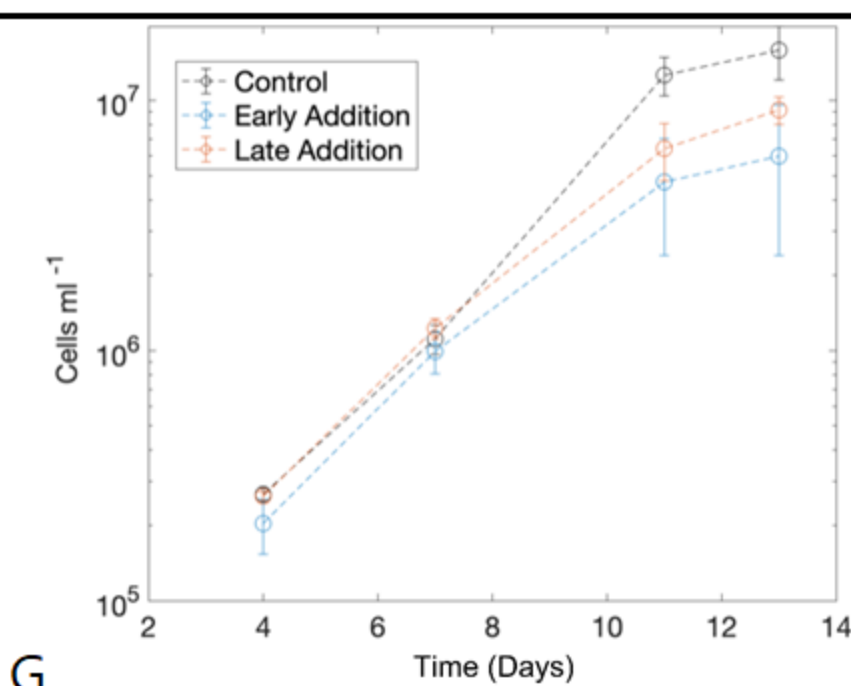

Supplement: FIG S1 [file mbio.01091-21-sf001.pdf]

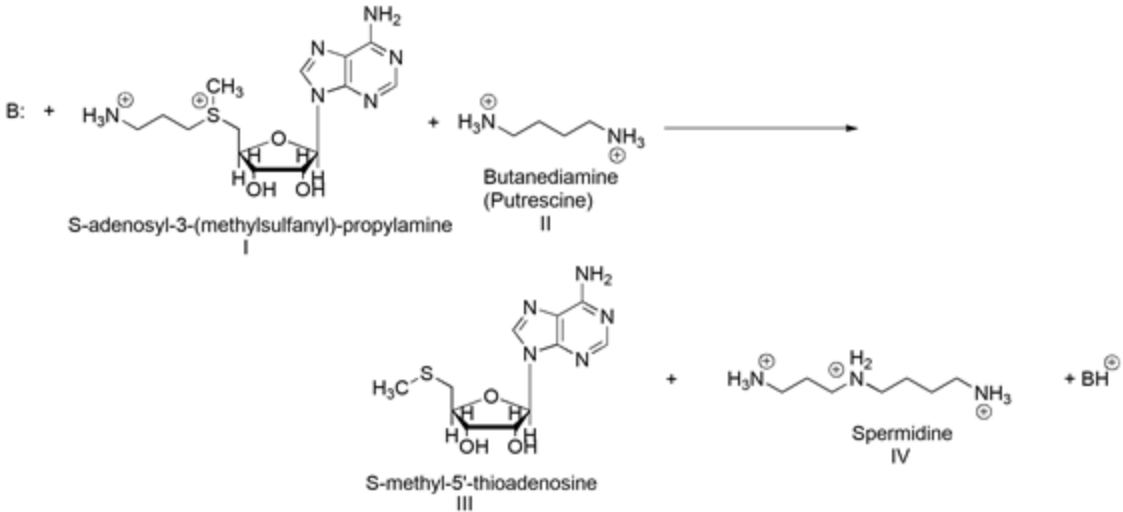

Supplement: FIG S3 [file mbio.01091-21-sf003.pdf]

A

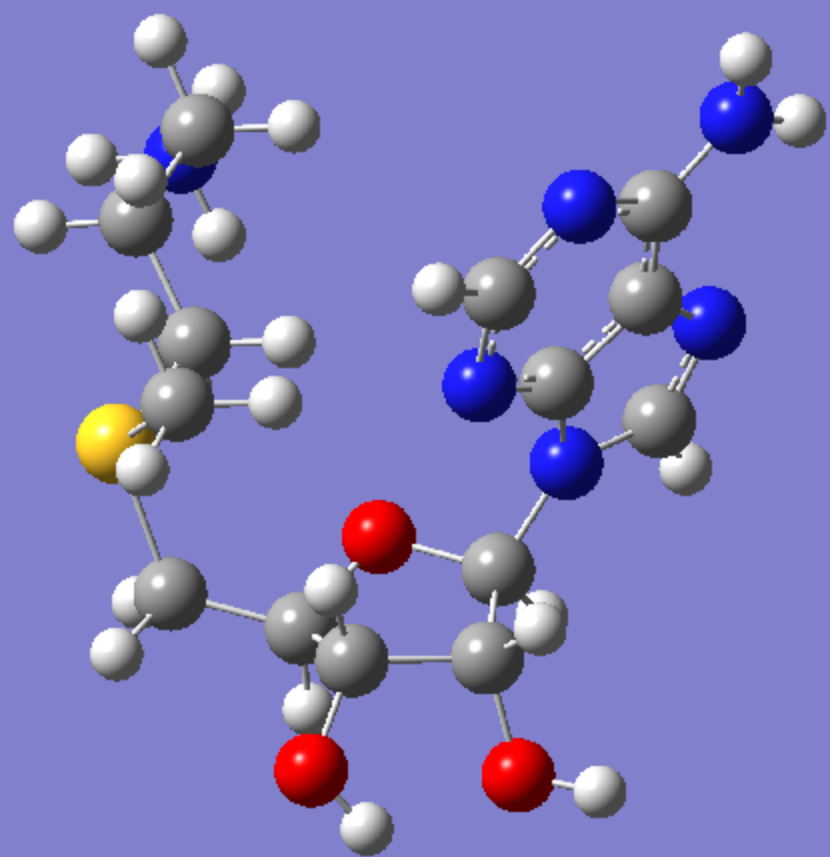

B

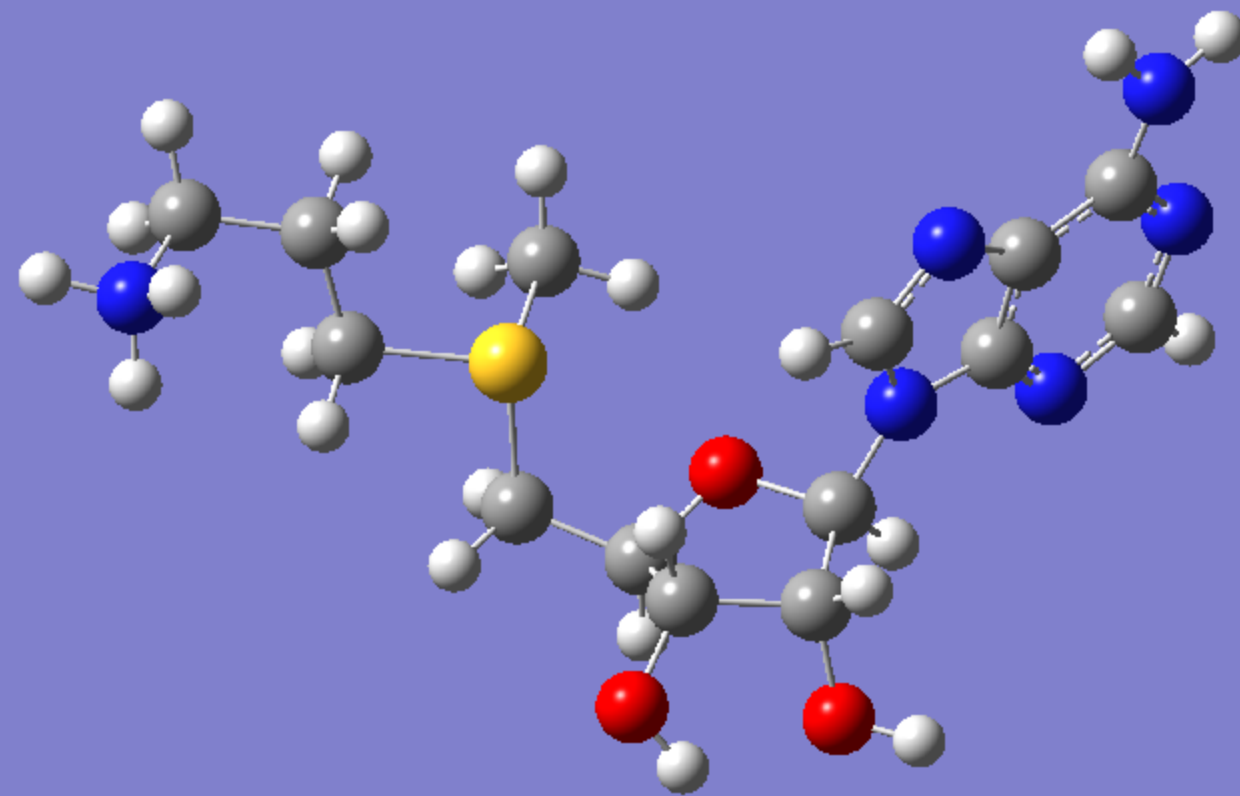

C

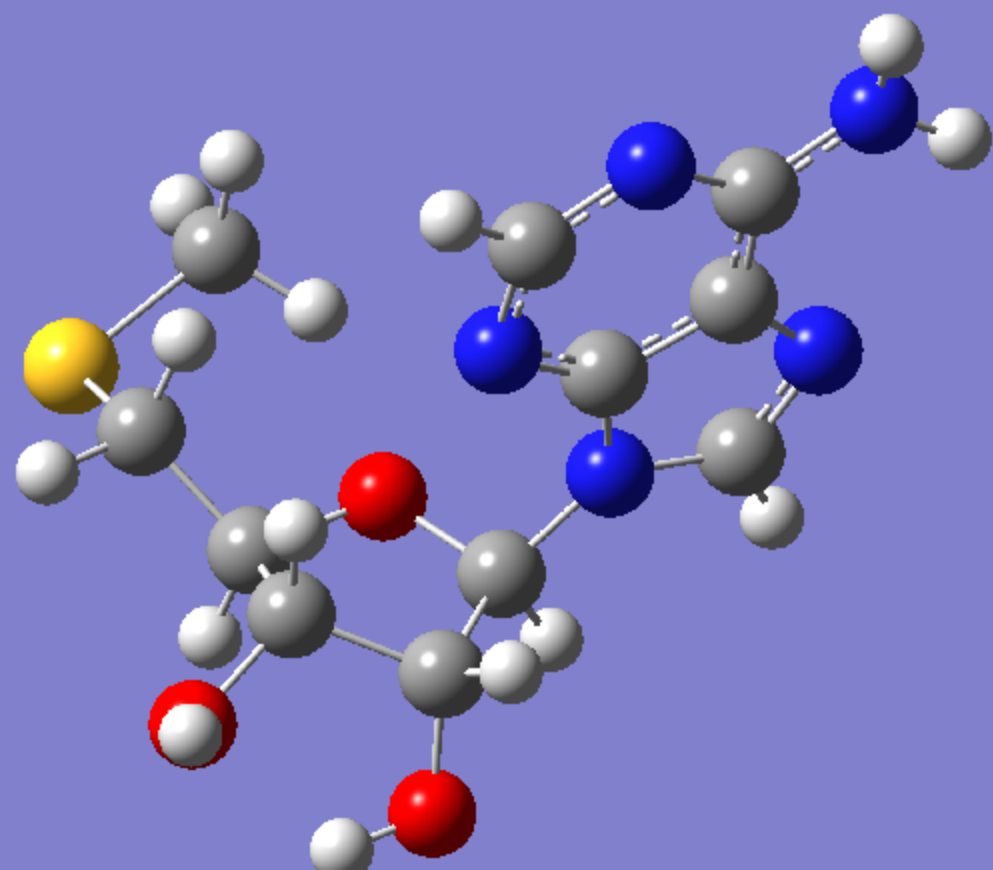

D

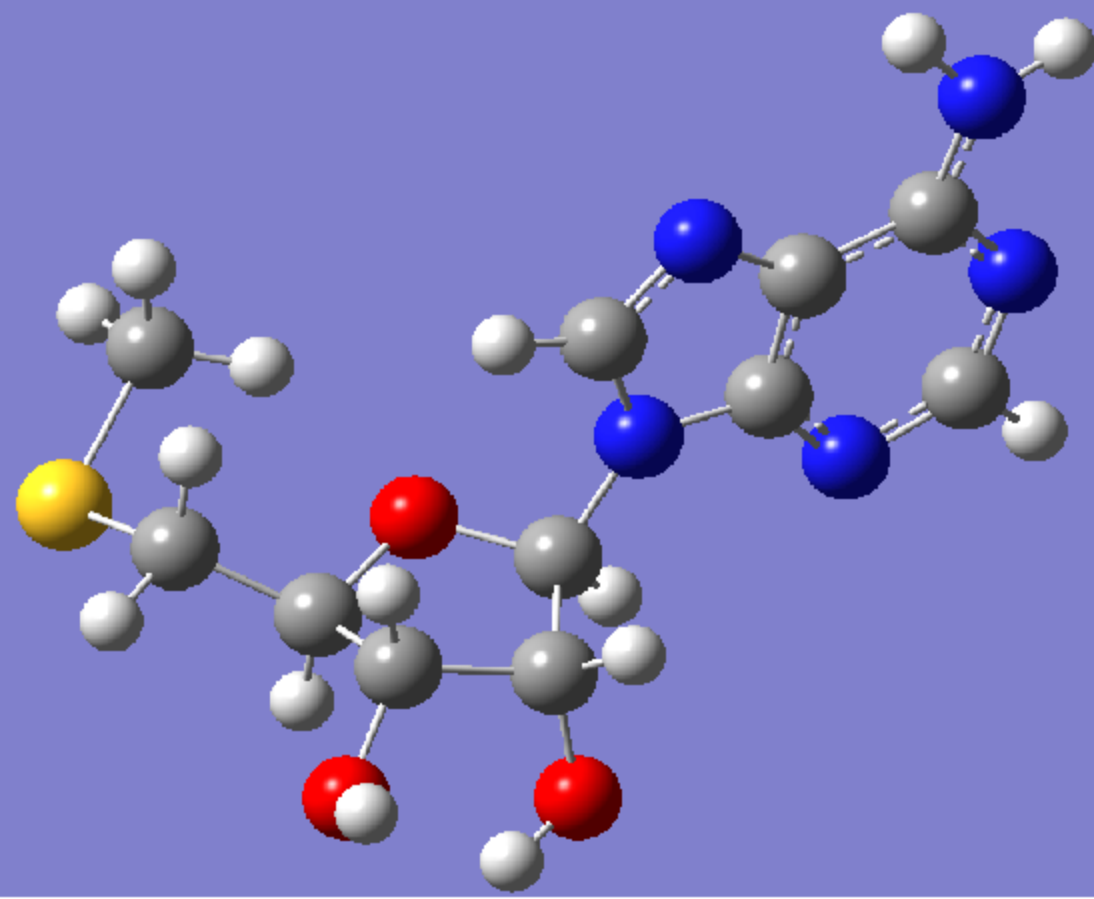

Supplement: FIG S4 [file mbio.01091-21-sf004.pdf]
