# Supplementary material for: SAR11 Cells Rely on Enzyme Multifunctionality To Metabolize a Range of Polyamine Compounds
Source: mBio. 2021 Aug 24;12(4):e01091-21. doi: 10.1128/mBio.01091-21 (PMC8437039; doi:10.1128/mBio.01091-21)
Supplement: FIG S2 [file mbio.01091-21-sf002.pdf]

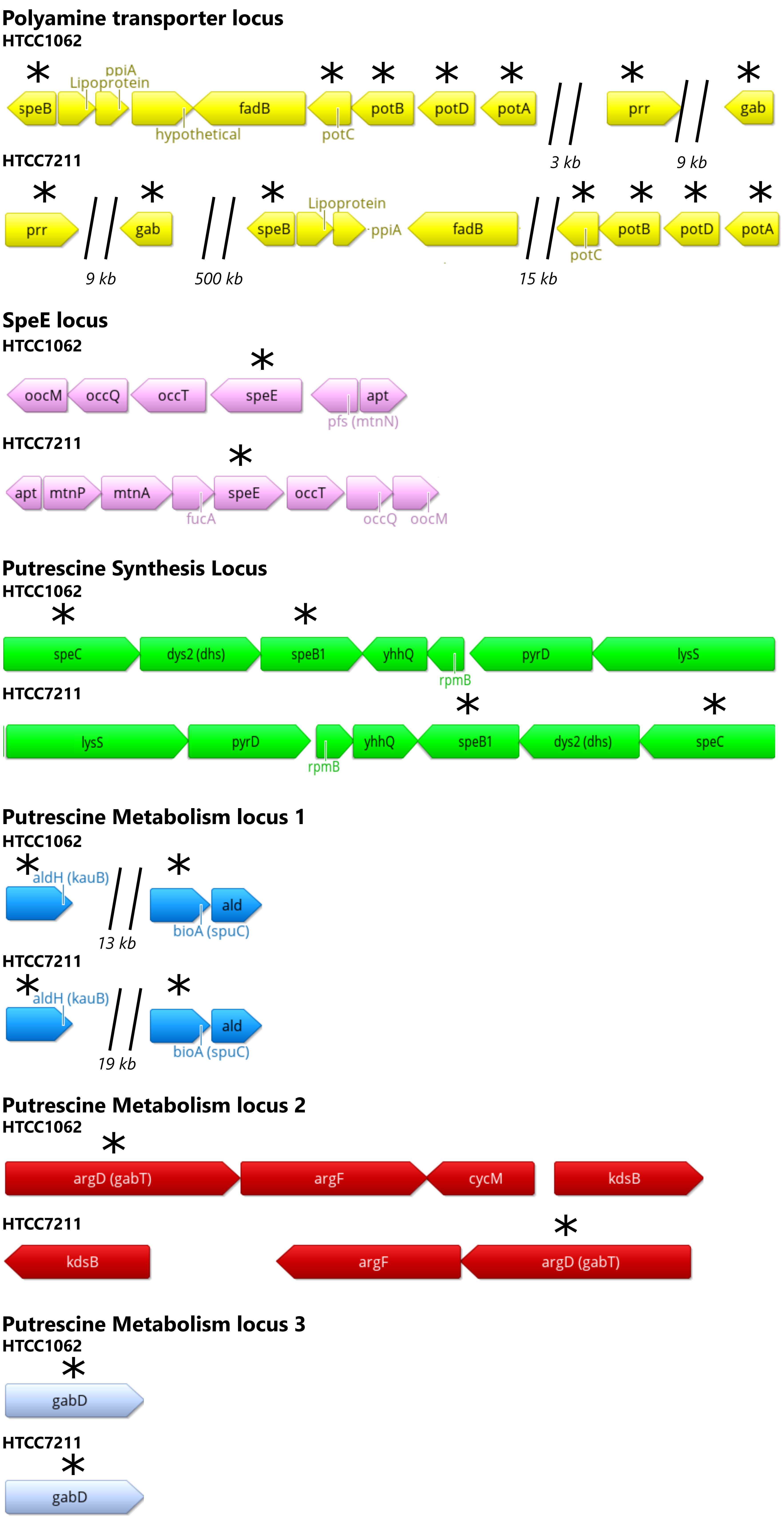

Polyamine transporter locus
HTCC1062
speB
Lipoprotein
ppiA
hypothetical
fadB
potC
potB
potD
potA
3 kb
prr
9 kb
gab
HTCC7211
prr
9 kb
gab
500 kb
speB
Lipoprotein
ppiA
fadB
15 kb
potC
potB
potD
potA
SpeE locus
HTCC1062
oocM
occQ
occT
speE
pfs (mtnN)
apt
HTCC7211
apt
mtnP
mtnA
fucA
speE
occT
occQ
oocM
Putrescine Synthesis Locus
HTCC1062
speC
dys2 (dhs)
speB1
yhhQ
rpmB
pyrD
lysS
HTCC7211
lysS
pyrD
rpmB
yhhQ
speB1
dys2 (dhs)
speC
Putrescine Metabolism locus 1
HTCC1062
aldH (kauB)
13 kb
bioA (spuC)
ald
HTCC7211
aldH (kauB)
19 kb
bioA (spuC)
ald
Putrescine Metabolism locus 2
HTCC1062
argD (gabT)
argF
cycM
kdsB
HTCC7211
kdsB
argF
argD (gabT)
Putrescine Metabolism locus 3
HTCC1062
gabD
HTCC7211
gabD
