# Supplementary material for: SAR11 Cells Rely on Enzyme Multifunctionality To Metabolize a Range of Polyamine Compounds
Source: mBio. 2021 Aug 24;12(4):e01091-21. doi: 10.1128/mBio.01091-21 (PMC8437039; doi:10.1128/mBio.01091-21)
Supplement: TABLE S2 [file mbio.01091-21-st002.docx]

Table S2 Maximum cell densities for SAR11 cultures grown with polyamine compounds as the sole nitrogen source with corresponding p-values. All p-values are from a one-sided t-test to assess whether the treatment had a significantly higher maximum cell density than the corresponding control. When an experimental treatment’s maximum cell density was lower than or equal to the corresponding control, a t-test was not conducted.

| Treatment | HTCC1062 | | | |
| --- | --- | --- | --- | --- |
|  | Max. cell density ± StDev (cells/mL) | p-value: vs +C, excess N | p-value: vs. +C, equimolar N | p-value: vs. -C |
| + Control,  Excess N | 4.8E6 ± 4.4E5 | - | - | - |
| + Control, Equimolar N | 0.61E6 ± 4.3E4 | - | - | - |
| - Control | 1.5E6 ± 4.6E5 | - | - | - |
| All Polyamines | 1.5E6 ± 7.8E5 | - | 0.10 | - |
| Putrescine (PUT) | 1.4E6 ± 1.3E6 | - | 0.25 | - |
| Cadaverine (CAD) | 0.98E6 ± 5.1E4 | - | 0.008 | - |
| Agmatine (AGM) | 0.30E6 ± 2.4E4 | - | - | - |
| Norspermidine (NSD) | 1.0E6 ± 7.1E5 | - | 0.12 | - |
| Spermidine (SPD) | 2.0E6 ± 7.4E5 | - | 0.05 | 0.23 |
| **Treatment** | **HTCC7211** | | | |
|  | Max. cell density ± StDev (cells/mL) | p-value: vs +C, excess N | p-value: vs. +C, equimolar N | p-value: vs. -C |
| + Control,  Excess N | 1.1E6 ± 8.4E4 | - | - | - |
| + Control, Equimolar N | 0.66E6 ± 1.4E5 | - | - | - |
| - Control | 0.87E6 ± 2.1E5 | - | - | - |
| All Polyamines | 0.26E6 ± 2.3E4 | - | - | - |
| Putrescine (PUT) | 1.3E6 ± 3.6E5 | 0.26 | 0.03 | 0.10 |
| Cadaverine (CAD) | 1.3E6 ± 2.1E5 | 0.09 | 0.005 | 0.03 |
| Agmatine (AGM) | 0.35E6 ± 4.9E4 | - | - | - |
| Norspermidine (NSD) | 0.22E6 ± 3.4E4 | - | - | - |
| Spermidine (SPD) | 1.5E6 ± 1.6E4 | 0.002 | 0.0003 | 0.004 |
