# Supplementary material for: SAR11 Cells Rely on Enzyme Multifunctionality To Metabolize a Range of Polyamine Compounds
Source: mBio. 2021 Aug 24;12(4):e01091-21. doi: 10.1128/mBio.01091-21 (PMC8437039; doi:10.1128/mBio.01091-21)
Supplement: TABLE S3 [file mbio.01091-21-st003.docx]

Table S3 Intracellular polyamine measurements from fingerprinting experiments (see Figure 2) fall within the error range of corresponding extracellular footprint measurements from the spent culture media. The intracellular polyamine levels in cells grown on polyamines were back calculated to mole values based on the number of cells in the culture when harvested. The standard deviation (stdev) for the extracellular measurements of polyamine compounds from the corresponding spent media were also back calculated to mole values for comparison. The only compound that clearly falls outside the error measurements is spermidine in HTCC7211.

| Compound | HTCC1062 | | HTCC7211 | | |
| --- | --- | --- | --- | --- | --- |
|  | Intracellular level (pmoles) | StDev of extracellular measurement (pmoles) | | Intracellular level (pmoles) | StDev of extracellular measurement (pmoles) |
| Putrescine (PUT) | 2.74 | 170 | | 50.5 | 114 |
| Cadaverine (CAD) | 0.04 | 278 | | 1.91 | 417 |
| Agmatine (AGM) | 0.09 | 58.4 | | 18.2 | 51.8 |
| Norspermidine (NSD) | 0.11 | 291 | | 12.7 | 39.7 |
| Spermidine (SPD) | 14.0 | 264 | | 239 | 83.3 |
