# Supplementary material for: SAR11 Cells Rely on Enzyme Multifunctionality To Metabolize a Range of Polyamine Compounds
Source: mBio. 2021 Aug 24;12(4):e01091-21. doi: 10.1128/mBio.01091-21 (PMC8437039; doi:10.1128/mBio.01091-21)
Supplement: TABLE S4 [file mbio.01091-21-st004.docx]

Table S4 Computed energies for the compounds of interest in the spermidine synthase reaction (Figure S3). I: S-adenosyl-3-(methylsulfanyl)-propylamine; III: S-methyl-5’-thioadenosine.

| **Compound** | **Conformer** | **E, Hartrees** |
| --- | --- | --- |
| I | “Compact” | -1500.073162 |
| I | “Extended” | -1500.075329 |
| III | “Compact” | -1325.910180 |
| III | “Extended” | -1325.913333 |
| Putrescine |  | -270.077240 |
| Spermidine |  | -443.819954 |
| Water |  | -76.426854 |
| Hydronium Ion |  | -76.823311 |
| Imidazole |  | -226.24186 |
| Imidazolium Ion |  | -226.69858 |
