# Supplementary material for: SAR11 Cells Rely on Enzyme Multifunctionality To Metabolize a Range of Polyamine Compounds
Source: mBio. 2021 Aug 24;12(4):e01091-21. doi: 10.1128/mBio.01091-21 (PMC8437039; doi:10.1128/mBio.01091-21)
Supplement: TABLE S5 [file mbio.01091-21-st005.docx]

Table S5 Estimated energies of reaction for the canonical spermidine synthase reaction (Figure S3) using either water or imidazole as the proton acceptor. It is expected that ΔH° values will be quite similar to ΔE values.

| **Proton Acceptor** | **ΔE, Hartree** | **ΔE, kcal/mol** | **pK_a_ of BH^+^** |
| --- | --- | --- | --- |
| Water | 0.022825 | 14.32 | -1.70 |
| Imidazole | -0.037438 | -23.49 | 6.95 |
