# Supplementary material for: SAR11 Cells Rely on Enzyme Multifunctionality To Metabolize a Range of Polyamine Compounds
Source: mBio. 2021 Aug 24;12(4):e01091-21. doi: 10.1128/mBio.01091-21 (PMC8437039; doi:10.1128/mBio.01091-21)
Supplement: TEXT S1 [file mbio.01091-21-s0001.docx]

**Text S1**

To explore the hypothesis that the SAR11 spermidine synthase enzyme is capable of catalyzing reactions that are the reverse of its ordinary action of polyamine formation (Figure S3), we explored thermodynamic models for the reaction catalyzed by spermidine synthase. The first step of the approach was to explore conformational space for compounds I and III (putrescine and spermidine were modeled in their fully extended forms and no conformational search was explored; Figure S3). In each case, multiple conformations were found within a 5 kcal/mol range, and two each were taken for DFT studies. One of the conformers mimicked the more “extended” form seen as the bound form in crystal structures of spermidine synthase (http://doi.org/10.2210/pdb3C6K/pdb); the other was a more “compact” form that was the global minimum, differing largely in the orientation of the adenine ring relative to the ribose (Figure S4). In the case of compound **I**, S-adenosyl-3-(methylsulfanyl)-propylamine, the “Extended” conformer is actually the global minimum by about 1 kcal/mol (Table S4). The difference is larger for **III**, S-methyl-5’-thioadenosine (appr. 2 kcal/mol) and now the “Compact” form is preferred (Table S4). In all likelihood the steric congestion of the alkylated sulfur in **I** dominated, whereas some van der Waals attraction between sulfur and the adenine aromatic system may be responsible for the stabilization of the “Compact” conformer in **III**. Of course, this latter observation may be an artifact of using the B3LYP functional; this is a known issue with that functional.

Reaction energies were estimated using the minimum energy conformer for each compound (Table S5). As can be seen, the reaction energy is highly dependent on the nature of the proton acceptor. We initially considered that release of the proton to the cellular medium would make water the likely terminal proton acceptor. Observing the relatively large positive ΔE (it is expected that ΔH° values will be quite similar to ΔE values), we considered that other proton acceptors are present; we chose to look at imidazole as a model for deprotonation by a histidine residue. This had a major impact by reversing the sign of ΔE and now favoring the reaction.

This is perhaps not surprising if we analyze several thermodynamic components in the reaction. The fundamental reaction is an exchange of a C-S bond (average BDE 65 kcal/mol) for a C-N bond (average BDE 73 kcal/mol), net exergonic by 8 kcal/mol ((a) T. L. Cottrell, *The Strengths of Chemical Bonds*, 2d ed., Butterworth, London, (1958); (b) B. deB. Darwent, *National Standard Reference Data Series*, National Bureau of Standards, no. 31, Washington, (1970). (c) S. W. Benson, *J. Chem. Educ*. **1965**, *42,* 502. (d) J. A. Kerr, *Chem. Rev*. **1966**, *66,* 465). However, this ignores charge reorganization, possible changes in H-bonding, conformational changes, and importantly, stabilization of the proton generated in the reaction. The latter is significant and will vary based on the pK_a_ of the conjugate acid being formed. The stronger the base strength of the conjugate base (the weaker the conjugate acid strength of the proton carrier), the more this reaction will favor the products. We can note that the availability of proton carriers of varying base strength in a cellular system suggests a high degree of tunability for the directionality of this reaction.
