# Supplementary material for: SAR11 Cells Rely on Enzyme Multifunctionality To Metabolize a Range of Polyamine Compounds
Source: mBio. 2021 Aug 24;12(4):e01091-21. doi: 10.1128/mBio.01091-21 (PMC8437039; doi:10.1128/mBio.01091-21)
Supplement: TABLE S1 [file mbio.01091-21-st001.docx]

Table S1 Recoveries for each of the polyamine compounds using the two extraction methods used. Extracellular recovery is the recovery percentage of standards extracted from artificial seawater (ASW) media using solid phase extraction (SPE). 500 nM standards of each compound were added to 10 mL ASW and extracted as described in the Methods. Reported values are the average and standard deviation of triplicate samples. For the intracellular recovery, 125 nM standards of each compound in 10 µL volume were extracted using the intracellular extraction method described in the Methods. Reported values are the average and standard deviation of duplicate samples.

| Compound | Extracellular recovery  ± standard deviation | Intracellular recovery  ± standard deviation |
| --- | --- | --- |
| Putrescine (PUT) | 86.8 ± 25.1 | 58.2 ± 13.0 |
| Cadaverine (CAD) | 88.6 ± 13.2 | 74.6 ± 10.5 |
| Agmatine (AGM) | 49.7 ± 9.73 | 119.0 ± 30.3 |
| Norspermidine (NSD) | 101.6 ± 4.49 | 62.1 ± 10.1 |
| Spermidine (SPD) | 89.7 ± 3.96 | 64.6 ± 9.10 |
